# Machine learning models based on immunological genes to predict the response to neoadjuvant therapy in breast cancer patients

**DOI:** 10.3389/fimmu.2022.948601

**Published:** 2022-07-22

**Authors:** Jian Chen, Li Hao, Xiaojun Qian, Lin Lin, Yueyin Pan, Xinghua Han

**Affiliations:** ^1^ Department of Oncology, The First Affiliated Hospital of USTC, Division of Life Sciences and Medicine, University of Science and Technology of China, Hefei, China; ^2^ Clinical Research Center for Cancer Bioimmunotherapy of Anhui Province, Hefei, China

**Keywords:** machine learning, immunological gene, neoadjuvant therapy, pathological complete response, breast cancer

## Abstract

Breast cancer (BC) is the most common malignancy worldwide and neoadjuvant therapy (NAT) plays an important role in the treatment of patients with early BC. However, only a subset of BC patients can achieve pathological complete response (pCR) and benefit from NAT. It is therefore necessary to predict the responses to NAT. Although many models to predict the response to NAT based on gene expression determined by the microarray platform have been proposed, their applications in clinical practice are limited due to the data normalization methods during model building and the disadvantages of the microarray platform compared with the RNA-seq platform. In this study, we first reconfirmed the correlation between immune profiles and pCR in an RNA-seq dataset. Then, we employed multiple machine learning algorithms and a model stacking strategy to build an immunological gene based model (Ipredictor model) and an immunological gene and receptor status based model (ICpredictor model) in the RNA-seq dataset. The areas under the receiver operator characteristic curves for the Ipredictor model and ICpredictor models were 0.745 and 0.769 in an independent external test set based on the RNA-seq platform, and were 0.716 and 0.752 in another independent external test set based on the microarray platform. Furthermore, we found that the predictive score of the Ipredictor model was correlated with immune microenvironment and genomic aberration markers. These results demonstrated that the models can accurately predict the response to NAT for BC patients and will contribute to individualized therapy.

## Introduction

Breast cancer (BC) is the most common malignant tumor and the fifth leading cause of cancer-related death worldwide. In 2020, about 2,261,419 females were diagnosed with BC, and 684,996 died from BC globally (1). Neoadjuvant therapy (NAT) plays an important role in multidisciplinary treatment for early and locally advanced BC. The latest study has demonstrated that residual cancer burden after NAT in BC patients is significantly associated with event-free survival (EFS) (2). Moreover, achieving pathological complete response (pCR) may indicate longer disease-free survival (DFS) and overall survival (OS) than others (3). Therefore, the current purpose of NAT is not only to downstage for surgery, but also to evaluate therapy response *in vivo* and improve prognosis. At present, NAT for BC patients with aggressive phenotypes has been recommended by several clinical practice guidelines. To achieve higher pCR rates and better prognosis, anthracycline and/or taxane-based therapy is currently the preferred treatment regimen. However, only 14.7%~52.9% (4) patients who undergo NAT will achieve pCR and have favorable outcomes, others, if initially operable, may not get additional benefits from NAT, but may suffer from side effects or even disease progression that may adversely affect the surgery. Therefore, it is crucial and necessary to predict the response to NAT to optimize the treatment plan.

In recent years, the development of artificial intelligence has promoted the progress of precision medicine. It can help clinicians gain insight into complex and large medical data to make more accurate decisions and to improve the outcomes for patients. For example, an intelligent VAB (5), which is a machine learning algorithm based on clinicopathological, image, and biopsy features, can identify BC patients who have achieved pCR after NAT, and exempt them from surgery. For drug response prediction, many artificial intelligence models have been reported, such as Deep Drug Response (6), DeepDR (7), tCNNS (8), DeepTTA (9), and VAE model (10). Although some complex and state-of-art algorithms were employed in these models, however, they were trained on an *in vitro* cell line or a pan-cancer dataset. This may lead to their prediction failure *in vivo* or in patients with specific cancer types and specific treatments due to the complex and variable biological environments *in vivo*. Moreover, a few machine learning models to predict the response to NAT in BC patients have also been proposed. Some models integrated clinicopathological features (11, 12) to predict pCR, but clinicopathological features are difficult to accurately and adequately characterize the complex biological properties associated with drug response in tumors. Other researchers established models using radiomics features (13). However, due to the low reproducibility (14) of radiomics features acquired through imaging, manual or semi-automatic segmentation, and mathematical extraction, these models are difficult to be widely used in clinical practice. Compared with the above two types of models, there are more prediction models based on gene expression levels that were determined by microarray (15–20). However, RNA-seq has replaced microarray as the dominant high-throughput gene expression level detection platform in recent years. Compared with microarray, RNA-seq is more accurate because of the absence of background level caused by cross-hybridization and wider dynamic detection range, more reproducible for its quantification by a direct digital other than an analogical manner, more feasible in clinical practice for its higher sensitivity and less sample required (21). Therefore, the model based on gene expression levels determined by the RNA-seq platform may be more robust and generalized. But to our knowledge, the RNA-seq platform-based model to predict pCR with NAT in BC patients has not been reported yet.

On the other hand, the relationship between the tumor immune microenvironment and the response to NAT in BC patients was observed more than a decade ago. Takayuki et al. (22) found immune-related pathways or gene sets were associated with the response to NAT in estrogen receptor-positive BC patients. Carsten et al. (23) discovered that the intratumoral lymphocyte was an independent predictor for pCR in BC patients who received NAT. Yasmin et al. (24) confirmed that lymphocyte-predominant BC and stromal lymphocytes were independent predictors for pCR in a prospective study. Besides lymphocytes, the expression of immunologic genes was also connected to the response to NAT in BC patients (25).

Therefore, in this study, we first analyzed the association between the immune microenvironment and the response to NAT in BC patients in an RNA-seq dataset. Then, we built machine learning models to predict the responses to NAT based on immunological genes in the RNA-seq dataset and validated the models in independent external datasets. Finally, we further analyzed the correlation between the model prediction and the immune microenvironment and genomic aberration.

## Materials and methods

### Data collection

Immune gene list and category information were obtained from IMMPORT (https://www.immport.org/). Transcripts per million (TPM) data and clinicopathological information of GSE163882 and GSE123845 datasets were obtained from Gene Expression Omnibus (GEO, https://www.ncbi.nlm.nih.gov/geo/) and the literature (26). GSE163882 was sequenced on Illumina NextSeq 500 platform and served as a training set in this study. GSE123845 was sequenced on Illumina HiSeq 2500 platform and served as an independent external test set in this study. The detailed information on the training and test datasets was described in **Table S1**. Raw sequencing data of GSE163882 were downloaded from Sequence Read Archive (SRA, https://www.ncbi.nlm.nih.gov/sra, Accession: PRJNA688066). Raw data and clinicopathological information of GSE20271 profiled on a DNA microarray platform (Affymetrix Human Genome U133A 2.0 Array), were obtained from GEO. Samples in the datasets with incomplete pCR information were removed. Another RNA-seq dataset of BC patients was downloaded from The Cancer Genome Atlas (TCGA, https://portal.gdc.cancer.gov/, Accession: TCGA-BRCA), and the relevant mutation and immune microenvironment data were obtained from the literature (27). Hallmark and Kyoto Encyclopedia of Genes and Genomes (KEGG) pathway gene sets were collected from MSigDB (https://www.gsea-msigdb.org/).

### Data preprocessing and model construction

All patients in the datasets had complete pCR information and none was excluded (**Table S1**). In total, 1,087 immunological genes were available across all datasets. Because TPM quantification was affected by the library size, we used a relative gene expression level, which was calculated as the proportion of the expression of an immunological gene to the expression of all immunological genes, to make the measurements of gene expression more comparable between samples across datasets. Before building the model, the Spearman rank correlation test was used for feature selection. Categorical variable features were encoded by one-hot encoding. The training set and test set were standardized by Z-score according to the mean and standard deviation of the training set. Principal component analysis (PCA) was used for dimensionality reduction. All the feature engineering was performed only on the training set and then applied on the testing set to avoid data leakage. For model development, a model stacking strategy (28) was used. Base models were trained on the origin training dataset and meta models were trained based on the outputs of the base models (**Figure S1**). Nine machine learning algorithms, including least absolute shrinkage and selection operator (Lasso) regression (29), ridge regression (RR) (30), elastic net regression (ENR) (31), support vector machine (SVM) (32), random forest (RF) (33), light gradient boosting machine (lightGBM) (34), fully-connected neural network (35) with one hidden layer (NNet1), fully-connected neural network with two hidden layers (NNet2), and fully-connected neural network with three hidden layers (Nnet3), were used for training the candidate base and meta models (**Figure S1**). The final outputs of the meta models were defined as prediction score (PS). Due to the imbalance of the ratio between the pCR patients and non-pCR patients in the training set, we adjusted the loss functions using class weights according to the class ratio when training the models. The hyperparameters of the models were optimized using Bayesian optimization through stratified 5-fold cross-validation in the training set according to the area under receiver operator characteristic curve (AUROC).

### Bioinformatics and statistical analysis

All computations and analyses in this study were implemented in Ubuntu 20.04.2 LTS using Python 3.8.12 and R 4.0.5. Detailed software and library information is listed in **Table S2**. In brief, raw RNA-seq data of GSE163882 were quality controlled and trimmed by TrimGalore, aligned by HISAT2 (36), and counted by featureCounts (37). Differential expression genes (DEGs) were identified using edgeR (38), DESeq2 (39), and limma (40). Over-representation analysis was performed using clusterProfiler (41). GSVA was used for single-sample gene set enrichment analysis (ssGSEA) (42). ImmuneSubtypeClassifier was used for the classification of immune subtypes (27). The immune and stromal cell sores were calculated by estimate (43). The abundance of immune cell subgroups was estimated using CIBERSORTx (44). scikit-learn and lightgbm were used for building models, and scikit-optimize was used for hyperparameters optimization. The genomic index (GGI) (45), Oncotype DX scores (46), and MammaPrint scores (47) for patients in the GSE20271 dataset were calculated by genefu (48). Brier score, area under precision-recall curve (AUPRC), AUROC, specificity (SPE), sensitivity (SEN), negative predictive value (NPV), and positive predictive value (PPV) were calculated by ModelMetrics. The 95% confidence interval and standard deviation of the metrics were estimated through stratified bootstrap resampling (2000 replicates). Spearman rank correlation test was performed to examine the associations. Fisher exact test and Mann-Whitney U-test or Kruskal-Wallis test were employed to compare the categorical and continuous data in different groups respectively. Two-sided P or false discovery rate (FDR) <0.05 was considered to be statistically significant.

## Results

### Association between immune profiles and pCR

**Figure 1:**
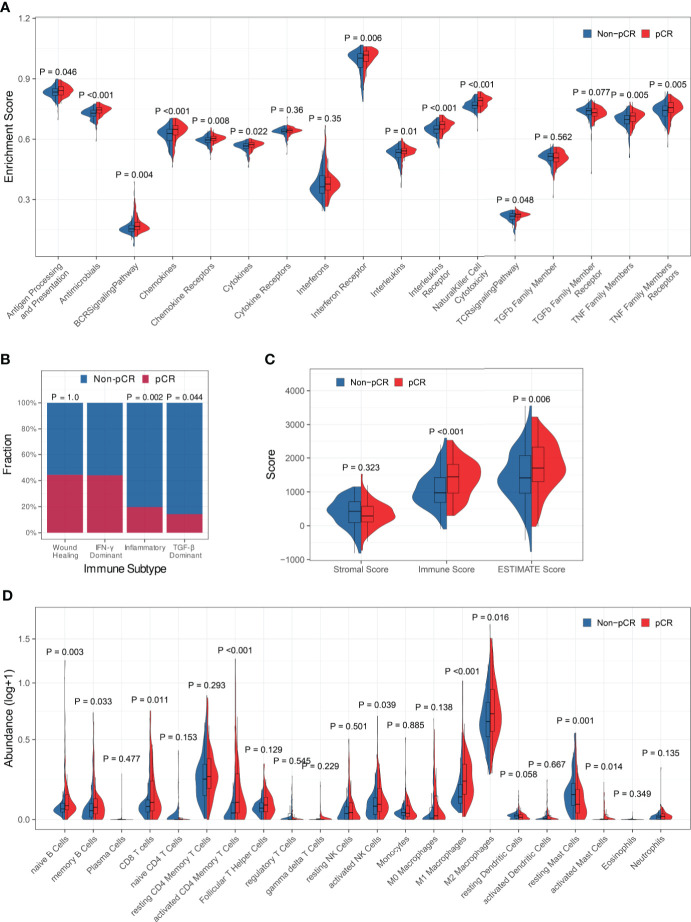
Association between immune profiles and pCR. **(A)** Comparisons of immune-related biological process enrichment scores between pCR and non-pCR patients. **(B)** Comparisons of pCR rates among patients with various immune subtypes. **(C)** Comparison of the level of stromal cell present, immune cell infiltration, and tumor purity between pCR and non-pCR patients. **(D)** Comparisons of infiltrating immune cell subsets between pCR and non-pCR patients.

Firstly, the association between immune profiles and pCR was analyzed in the training set. We employed three distinct methods (edgeR, DESeq2, and limma) to identify DEGs between patients who achieved pCR and those who did not (**Figures S2A-C**). There were 294 overlapping DEGs found, 133 of which were up-regulated and 161 of which were down-regulated (**Figure S2D**). Gene Ontology (GO) over-representation analysis revealed that the proteins encoded by the DEGs were the components of extracellular matrix, chromosome, and T cell receptors (**Figure S2E**). They were also involved in many cytokine-related biological functions (**Figure S2F**) and mainly participated in immune-related biological processes, especially lymphocyte-related (**Figure S2G**). KEGG pathway over-representation analysis demonstrated that the DEGs participated in immune-related signal pathways including viral protein interaction with cytokine and cytokine receptor, cytokine-cytokine receptor interaction, and chemokine signaling pathway (**Figure S2H**). ssGSEA demonstrated that the enrichment scores of antigen processing and presentation, antimicrobials, BCR signaling pathway, chemokines, chemokine receptors, cytokines, interferon receptor, interleukins, interleukins receptor, natural killer cell cytotoxicity, TCR signaling pathway, TNF family members, and TNF family members receptors were significantly increased in patients who achieved pCR (**Figure 1A**).

Then, we looked at the relationship between pCR and the immune subtypes and discovered that the pCR rate of IFN-γ dominant subtype patients was significantly high than that of inflammatory or TGF-b dominant subtype patients (**Figure 1B**). The immune scores and ESTIMATE scores for patients who achieved pCR were significantly higher than those for patients who did not achieve pCR, although there was no significant difference in stromal scores between them, indicating that patients who achieved pCR had increased immune cell infiltration and decreased tumor purity (**Figure 1C**). Furthermore, we compared immunological cell subsets in different patient groups and observed that the pCR patients exhibited increased infiltration of naïve B cells, memory B cells, CD8+ T cells, activated CD4+ memory T cells, activated NK cells, M1 and M2 macrophages, and activated mast cells (**Figure 1D**). All the above findings suggested that tumor immune profiles may be closely related to the response to NAT in BC patients.

### Model construction

Before building the model, we identified 62 immunological genes in the training set that were strongly associated with pCR by the Spearman rank correlation test (P < 0.001, **Figure S3A**). Then, we built two prediction models based on the 62 immunological genes and the 62 immunological genes combined with the clinicopathological characteristics (**Table S1**), respectively (hereafter called Ipredictor model and ICpredictor model respectively). For the Ipredictor model, we first standardized the training and test sets and implemented dimensionality reduction *via* PCA and kept 80% of the components (**Figure S3B**). After that, the number of input features was reduced to 23. Next, we trained nine candidate base models using different algorithms based on the original training data. The hyperparameters were optimized using Bayesian optimization through stratified 5-fold cross-validation in the training set (**Figure S4**), and the mean cross-validation AUROCs of the candidate base models were 0.749, 0.742, 0.748, 0.736, 0.709, 0.742, 0.718, 0.731, and 0.744 for the Lasso, RR, ENR, SVM, RF, lightGBM, NNet1, NNet2, and NNet3 base models respectively (**Table S3**). Because only when the base models are accurate and diverse can the stacked model perform better than any base model (49), we chose the Lasso, ENR, RR, lightGBM, and NNet3 base models, which are different types of models and whose mean AUROCs were greater than 0.74, as base models. Based on the predictions of the base models, we also trained nine candidate meta models using Bayesian optimization through stratified 5-fold cross-validation in the training set (**Figure S5**). The mean cross-validation AUROCs of the candidate meta models were 0.750, 0.751, 0.751, 0.752, 0.743, 0.741, 0.759, 0.755, and 0.752 for the Lasso, RR, ENR, SVM, RF, lightGBM, NNet1, NNet2, and NNet3 meta models respectively (**Table S3**). Most meta models were improved compared to the best base model. All neural network meta models (NNet1, NNet2, and NNet3) were superior to any linear meta model (Lasso, ENR, or RR). It may indicate that there may be some nonlinear relationship between the input features and labels that a linear model may not be sufficient to explain. Therefore, we chose the best candidate meta model NNet1, which was the simplest neural network and had the highest mean cross-validation AUROC, as the final meta model. For the ICpredictor model, clinicopathological characteristics which may be associated with the response to NAT, including estrogen receptor (ER) status, progesterone receptor (PR) status, human epidermal growth factor receptor 2 (HER2) status, stage, and histological grade were available in both the training set and test set (**Table S1**). However, about 27% of histological grade information and 33% of stage information were missing in the test set (**Table S1**). Therefore, we built the ICpredictor model by combining the ER, PR, and HER2 status with the Ipredictor model. Similar to the Ipredictor model development process, we first trained nine candidate base models (**Figure S6** and **Table S4**), then selected Lasso, ENR, RR, SVM, NNet1, NNet2, and NNet3 as base models for their mean AUROCs greater than 0.8, and then trained nine candidate meta models (**Figure S7**). But none of the candidate meta models was significantly improved compared with the base models (**Table S4**). According to Occam’s razor, considering the complexity and performance of the models, we choose the simplest Lasso base model as the final ICpredictor model. Therefore, the final Ipredictor model consisted of five base models (Lasso, ENR, RR, lightGBM, and NNet3 models) and one meta model (NNet1 model), and the final ICpredictor model was the lasso base model (**Figure S8**). In addition, for comparison, we also built a model only based on clinicopathological characteristics, including age, ER status, PR status, HER2 status, histological grade, and clinical stage, using the Lasso algorithm (hereafter called CPpredictor). The hyperparameter of the CPpredictor model was also optimized by Bayesian optimization through stratified 5-fold cross-validation in the training set.

### Model evaluation

After the models were constructed, we evaluated their predictive abilities. To compare their predictive abilities with those of clinicopathological characteristics, we imputed some missing clinicopathological information in the test set. It has been demonstrated that patients with a higher histological grade are more likely to achieve pCR in many studies (50–53). Some studies have also reported that patients with smaller tumor sizes and/or negative regional nodes are more likely to achieve pCR, although this is still controversial (50, 51). Therefore, we imputed the missing histological grade with Grade 3 if the patient achieved pCR and with Grade 1 if the patient did not. The clinical T stage was imputed with T1 for the completely missing one or earlier T stage for the undetermined one (for example T1-2) if the patient achieved pCR, and with T3/4 or more advanced stage if the patient did not achieve pCR. The regional lymph node status was imputed with negative for completely missing one or N0-1 if the patient achieved pCR, and with positive if the patient did not. These imputations gave the clinicopathological characteristics and the CPpredictor model maximum predictive abilities, which are greater than or equal to the actual predictive abilities.

**Figure 2:**
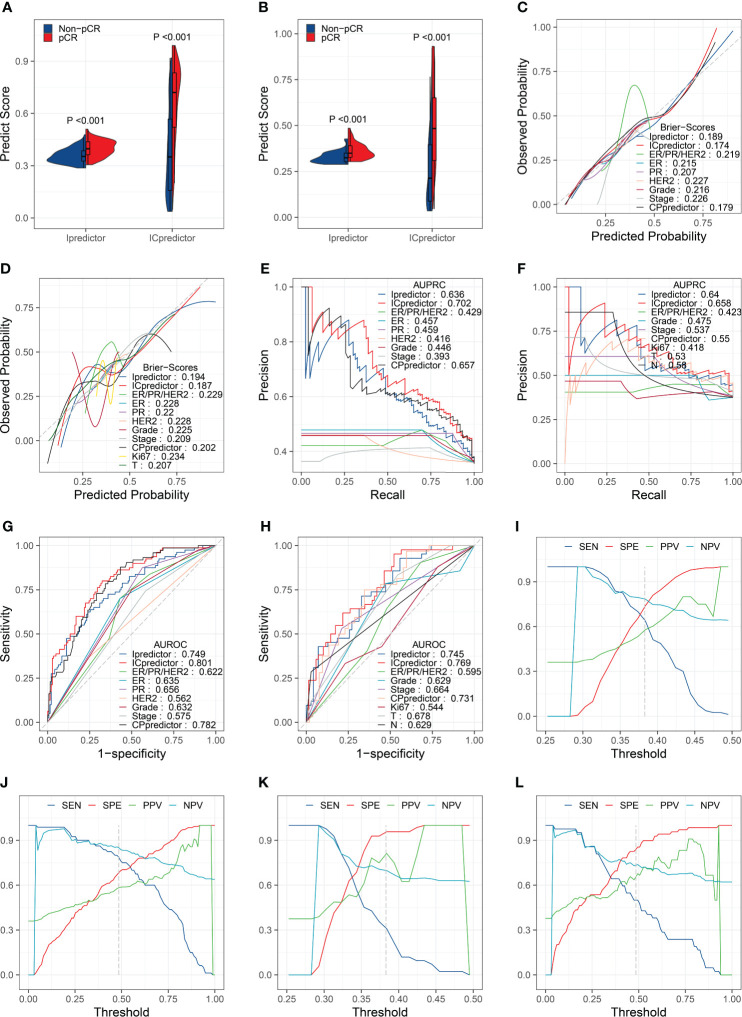
Model performance evaluation in the training and test sets. Comparisons of the PSs in pCR patients and non-pCR patients in the training set **(A)** and test set **(B)**. Brier scores of the models and clinicopathological characteristics in the training set **(C)** and test set **(D)**. AUPRCs of the models and clinicopathological characteristics in the training set **(E)** and test set **(F)**. AUROCs of the models and clinicopathological characteristics in the training set **(G)** and test set **(H)**. SENs, SPEs, PPVs, and NPVs for the Ipredictor **(I)** and ICpredictor **(J)** models in the training set. SENs, SPEs, PPVs, and NPVs for the Ipredictor **(K)** and ICpredictor **(L)** models in the test set.

The PSs for patients calculated by the Ipredictor and ICpredictor models in the training set and test set were significantly associated with the actual responses to NAT, and pCR patients had higher PSs (**Figures 2A, B**). The Brier scores of the Ipredictor and ICpredictor models were 0.189 and 0.174 in the training set (**Figure 2C** and **Table S5**), and were 0.194 and 0.187 in the test set (**Figure 2D** and **Table S5**). The AUPRCs of the Ipredictor and ICpredictor models were 0.636 and 0.702 in the training set (**Figure 2E** and **Table S6**), and were 0.64 and 0.658 in the test set (**Figure 2F** and **Table S6**). The AUROCs of the Ipredictor and ICpredictor models were 0.749 and 0.801 in the training set (**Figure 2G** and **Table S7**), and were 0.745 and 0.769 in the test set (**Figure 2H** and **Table S7**). These results suggested that, combined with ER/PR/HER2 status information, the predictive ability of the ICpredictor model was improved compared to the Ipredictor model. However, both the Ipredictor and ICpredictor models outperformed clinicopathological characteristics or the CPpredictor model in the test set, which is the main basis for screening for appropriate NAT candidates in current clinical practice.

Because the PS was a continuous value, we determine the optimal threshold value of the PS when the sum of SEN and SPE reached the maximum in the training set. According to the optimal threshold values, in the training set, the SPE, SEN, NPV, and PPV were 0.739, 0.637, 0.784, and 0.58 respectively for the Ipredictor model (**Figure 2I** and **Table S8**), and were 0.683, 0.787, 0.851, and 0.583 respectively for ICpredictor model (**Figure 2J** and **Table S8**); in the test set, the SPE, SEN, NPV, and PPV were 0.957, 0.31, 0.698, and 0.812 respectively for the Ipredictor model (**Figure 2K** and **Table S8**), and were 0.841, 0.5, 0.734, and 0.656 respectively for ICpredictor model (**Figure 2L** and **Table S8**). We also performed logistic regression analysis to access the independent predictive power of the models. Univariate analysis demonstrated that age, ER status, PR status, histological grade, CPpredictor PS, Ipredictor PS, and ICpredictor PS were related to pCR in the training set, and PR status, histological grade, clinical N stage, clinical T stage, clinical stage, CPpredictor PS, Ipredictor PS, and ICpredictor PS were related to pCR in the test set. Multivariate analysis suggested that both the Ipredictor PS and ICpredictor PS were the independent predictors for pCR in both the training set and test sets (**Table S9**).

**Figure 3:**
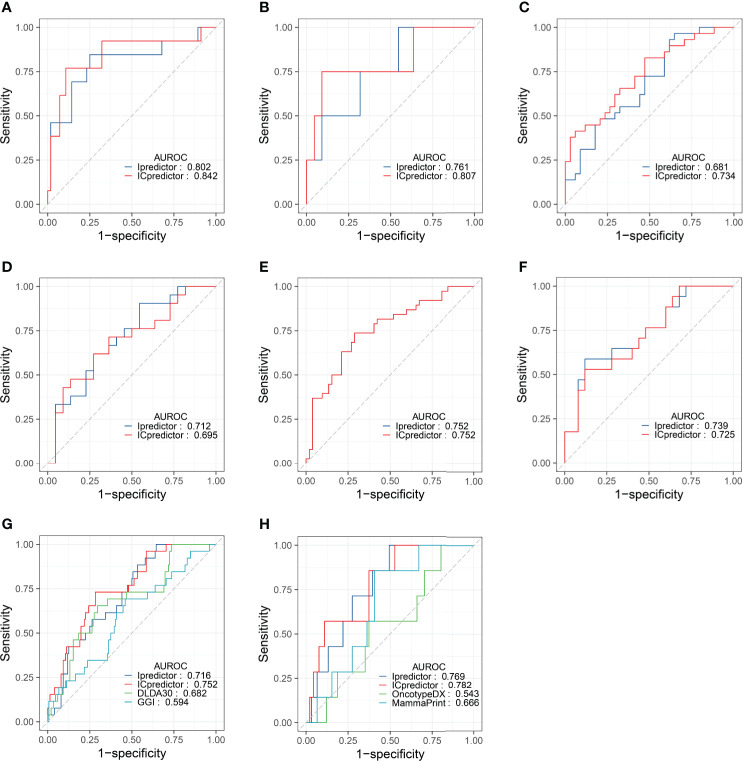
Model performance evaluation in the patient subgroups of the test set and GSE20271 dataset. AUROCs of the models for ER+/HER2- patients in the training **(A)** and test **(B)** set. AUROCs of the models for HER2+ patients in the training **(C)** and test **(D)** set. AUROCs of the models for ER-/HER2- patients in the training **(E)** and test **(F)** set. AUROCs of the models for all patients **(G)** and ER+/HER- patients **(H)** in the GSE20271 dataset.

Furthermore, we performed subgroup analyses. In ER+/HER2- patients, the AUROCs of the Ipredictor and ICpredictor models were 0.802 and 0.842 in the training set (**Figure 3A**), and were 0.761 and 0.807 in the test set (**Figure 3B**). In HER2+ patients, the AUROCs of the Ipredictor and ICpredictor models were 0.681 and 0.734 in the training set (**Figure 3C**), and were 0.712 and 0.695 in the test set (**Figure 3D**). In ER-/HER2- patients, the AUROCs of the Ipredictor and ICpredictor models were 0.752 and 0.752 in the training set (**Figure 3E**), and were 0.739 and 0.725 in the test set (**Figure 3F**). Other subgroup analysis results were listed in **Table S10**. These results demonstrated that the models can predict the pCR in most subgroups.

Although the Ipredictor and ICpredictor models were developed based on the RNA-seq platform dataset, we also calculated the relative gene expression levels, and attempted to evaluate and compare the model performance in the GSE20271 dataset, which is based on a microarray platform. The AUROCs for the Ipredictor and ICpredictor models in the GSE20271 dataset were 0.716 and 0.752 respectively, and superior to the previously proposed DLDA30 scores (15) and GGI (54), whose AUROCs were 0.682 and 0.594 respectively (**Figure 3G** and **Table S11**). Additionally, it has been reported that Oncotype DX scores (55) and MammaPrint scores (56) were significantly related to the response to NAT in ER+/HER2- BC patients. We also assessed and compared the predictive power of the models in the ER+/HER2- patients. The results showed that the AUROCs of the Ipredictor and ICpredictor models were 0.769 and 0.782 respectively, which also outperformed the Oncotype DX and MammaPrint scores, whose AUROCs were 0.543 and 0.666 respectively (**Figure 3H** and **Table S11**).

### Model exploration

**Figure 4:**
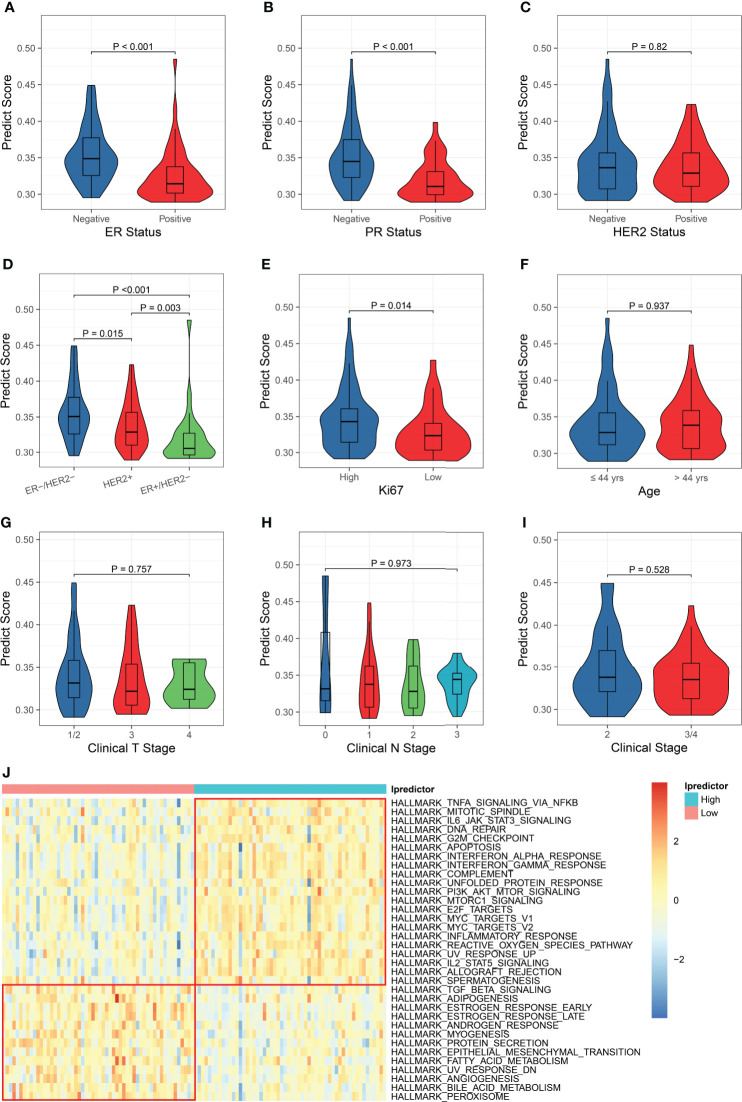
Clinicopathological and biological implications for the PS. The correlations between the PS and ER status **(A)**, PR status **(B)**, HER2 status **(C)**, ER/HER2 status **(D)**, Ki67 status **(E)**, age **(F)**, clinical T stage **(G)**, clinical N stage **(H)**, and clinical stage **(I)**. **(J)** Comparisons of the hallmark gene set enrichment scores between the patients with high and low PSs.

Since the Ipredictor model was developed based on molecular features, we further analyzed correlations between the PS and clinicopathological characteristics in the test set. As expected, the PS was significantly associated with ER/PR status. The ER- and/or PR- patients exhibited higher PSs, indicating more likely to achieve pCR (**Figures 4A, B**). Although the PS was not significantly correlated to HER2 status, the ER-/HER2- patients had the highest PSs, followed by the HER2+ patients, and the ER+/HER2- patients had the lowest PSs (**Figures 4C, D**). In addition, the PS was associated with Ki67 status (**Figure 4E**), whereas no significant correlations were found between the PS and age, clinical T stage, clinical N stage, and clinical stage (**Figures 4F–I**).

To explore the biological implications of the model, we conducted KEGG pathway and GO molecular function over-representation analyses on the 62 immunological genes that were significantly associated with pCR and were used for building models. The results revealed that these genes were involved not only in immune-related signaling pathways, but also in HIF-1, JAK-STAT, MAPK, apoptosis, and some cancer-related signaling pathways (**Figure S9A**), They also played a role in many growth factor bindings and protein kinase activity (**Figure S9B**). Then, we calculated the enrichment scores using the ssGSEA algorithm based on the hallmark and KEGG pathway gene sets and compared the enrichment scores of patients with high and low Ipredictor PSs grouped according to the median PS. For the hallmark gene set enrichment analysis, the PS was significantly correlated to DNA repair, G2M checkpoint, apoptosis, PI3K/AKT/mTOR signaling pathways, E2F and MYC targets, epithelial-mesenchymal transition, and angiogenesis (**Figure 4J**); for the KEGG pathway gene set enrichment analysis, the PS was significantly associated with DNA repair, cell cycle, P53, VEGF, and some cancer-related signaling pathways (**Figure S10**). We also found that drug metabolism cytochrome P450 and ABC transporters pathways were more activated in patients with low PSs, which were associated with drug resistance (**Figure S10**).

**Figure 5:**
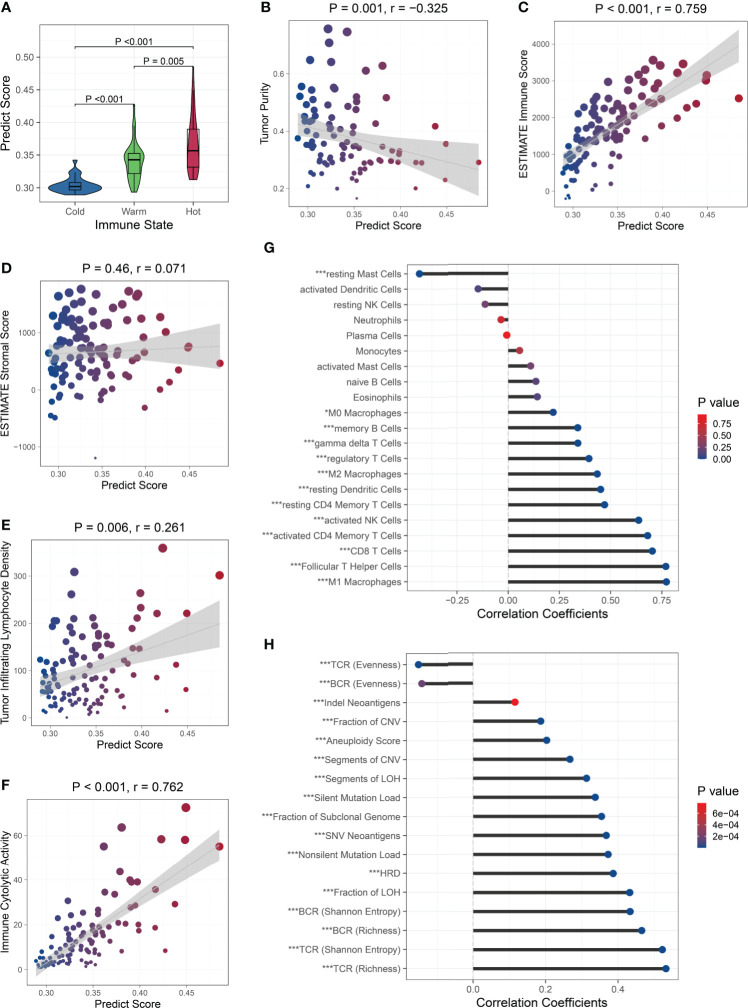
Relationship between the PS and immune microenvironment and genomic aberrations. The PS was associated with the immune state **(A)**, tumor purity **(B)**, and immune cell infiltration **(C)**, but was not with the stromal cell infiltration **(D)**. The PS was positively correlated to the TIL density **(E)** and cytolytic activity **(F)**. Association between the PS and the abundance of immune cell subsets **(G)** and genomic aberration markers **(H)**. ^∗^P < 0.05,^∗∗∗^P < 0.001.

Next, we analyzed the relationship between the PS and immune microenvironment. Firstly, we found that patients with ‘hot’ tumors had higher PSs than patients with ‘warm’ tumors, while patients with ‘cold’ tumors had the lowest PSs (**Figure 5A**). The PS was negatively correlated with the tumor purity (**Figure 5B**) and positively correlated with the immune cell infiltration (**Figure 5C**), but was not associated with the stromal cell infiltration (**Figure 5D**). Moreover, the PS was positively correlated to the tumor infiltrating lymphocyte (TIL) density (**Figure 5E**) and the cytolytic activity (**Figure 5F**). These results suggested that patients with higher PSs had lower tumor purity and more infiltrating immune cells, especially TILs. We further analyzed the correlation between the PS and immune cell subsets and found that the PS was positively associated with the abundance of all kinds of macrophages and T cells, memory B cells, resting dendritic cells, and activated NK cells, while negatively associated with the abundance of resting mast cells. There were no correlations between the PS and the abundance of naive B cells, plasma cells, resting NK cells, monocytes, activated dendritic cells, activated mast cells, eosinophils, and neutrophils (**Figure 5G**). These results indicated that the PS was mainly related to the lymphocytes, which are the main effector cells in the immune response to the tumor. We also analyzed the relationship between the PS and the expression of antigen presentation-related genes and immune checkpoint-related genes (27), and found that PS was positively correlated with the expression of most of them, including both checkpoint stimulator and inhibitor genes (**Figure S11**).

Because the immunological gene expression is associated with genomic aberrations in BC (57), we further investigated the relationship between the PS and genomic alternations in the TCGA BC patients. Firstly, among the BC-related driver genes (58), higher mutation frequencies of *TP53*, *NF1*, *RB1*, *PTEN*, and *CHD4* were observed in patients with high PSs, while higher mutation frequencies of *PIK3CA* and *MAP3K1* were observed in patients with low PSs (**Figure S12**). Then, we found the PS was significantly positively correlated to the silent and nonsilent mutation load, fraction and segment of copy number variation, fraction and segment of loss of heterozygosity (LOH), fraction of subclonal genome, homologous recombination deficiency (HRD), and aneuploidy score. The Indel neoantigens and somatic single-nucleotide variation (SNV) neoantigens induced by mutations were also significantly positively associated with the PS. The Shannon entropy and richness of BCR and TCR were positively correlated with PS, while the evenness of BCR and TCR was negatively correlated with the PS (**Figure 5H**). These results suggested that patients with high PSs carried more variations, generated more neoantigens, and showed greater BCR and TCR diversities.

## Discussion

In this study, we reconfirmed the correlation between immune microenvironment and pCR in BC patients receiving NAT in an RNA-seq dataset. Then, we built machine learning models to predict pCR with NAT for BC patients using immunological gene expression measured by the RNA-seq platform and validated the predictive power and robustness of the models in independent external datasets. Furthermore, we demonstrated that the model was related to the immune microenvironment and genomic mutations.

Initially, we found that most differentially expressed genes between the pCR and non-pCR patients were involved in immune-related pathways. Immune microenvironment analysis showed increased immune cell infiltrating in the pCR patients. Among the immune cell subsets, more lymphocytes, NK cells, and macrophages infiltrated in the pCR patients. These findings in the RNA-seq dataset were similar to the previous studies (22, 24).

We employed multiple machine learning algorithms and a model ensemble strategy to build the Ipredictor model based on the immunological genes determined by the RNA-seq platform. Then, we combined the Ipredictor model with the ER, PR, and HER2 status to build the ICpredictor model. In the independent external RNA-seq test set, the performance of the models outperformed clinicopathological characteristics. Subgroup analyses demonstrated that the models can also predict the response to NAT regardless of the HR/HER2 status, including patients with ER+/HER2-, who are generally considered to be insensitive to NAT.

Recently, several models based on gene expression detected by the microarray platform to predict pCR with NAT in BC patients have been proposed (15–20). As mentioned above, microarray quantifies gene expression in an analogous manner according to the brightness of the fluorescence signal for hybridization. Many factors may affect the brightness, such as differential scanner settings, different hybridization conditions, and different amounts of RNA hybridization in different arrays (59). Furthermore, due to the brightness detection sensitivity and hybridization saturation, some genes with low and high abundance can not be accurately measured. These disadvantages lead to more pronounced batch effects within and between samples. Therefore, a model based on unstable and inaccurate gene expression measurement may not be robust and generalized in practical application. More importantly, the major limitation is that some studies (17–20) used normalization or ComBat (60) before model building to reduce batch effects. However, these gene-wise scaling methods require to use the parameters of the entire test set, such as the mean and standard deviation of the test set, which may lead to data leakage. Moreover, if only one sample rather than a batch of samples needs to be predicted at a time in practice, the parameters of the single sample will not be available. This obviously means that the model pipeline and prediction cannot be implemented in a real clinical application scenario. Compare with these studies, we used a relative quantification to measure gene expression, which was similar to TPM. Because it is a relative quantification only focusing on the proportion of gene expression, it can be implemented across datasets. We also attempted to validate the model in a microarray dataset. The results demonstrated that both the Ipredictor and ICpredictor can predict the response to NAT in the GSE20271 dataset, and were superior to some other proposed models or markers.

Furthermore, we explored the association between the Ipredictor model and the clinicopathological characteristics, biological processes, immune microenvironment, and genomic abbreviations. Firstly, we found that ER-negative or Ki67-high patients had higher PSs and were more likely to achieve pCR. These were consistent with the literature (61). Then, enrichment analysis revealed that some cancer- and immune-related pathways and biological processes were more activated in patients with high PSs. Additionally, the cytochrome P450 metabolism and ABC transporter pathways were more activated in patients with low PSs. This could partially account for the low pCR rate in patients with low PSs. The PS was also positively correlated to the immunological gene expression and tumor infiltrating immune cells, even including immunosuppressive genes and regulatory T cells. These seemingly contradictory results, however, were consistent with the literature (18, 62) and the possible explanation was a feedback activation of immunosuppressive pathways. Finally, we also found the immunological gene-based PS was associated with SNV, CNV, and chrome abbreviations. This is likely due to more genomic alternations inducing stronger immune responses. These results indicated that the Ipredictor PS may also be a reflection of the immune microenvironment and genomic mutation status.

Finally, there are also some issues or limitations that should be noted in this study. Firstly, relatively high SPEs but low SENs for the models were observed in the test set. This means that the patients who are predicted to achieve pCR will probably achieve pCR, but the predicted non-pCR patients may not necessarily fail to achieve pCR. Thus, identifying candidates for NAT according to the models may improve the pCR rate and reduce unnecessary NAT, but may also miss some potential pCR patients. On the other hand, we also found that the SENs obviously decreased and the SPEs obviously increased in the test set compared with those in the training set. This may suggest that the optimal threshold values of the PSs in the test set were different from those in the training set, which may be associated with dataset shift induced by differences in genetic background, treatment, and clinicopathological characteristics between the datasets. The optimal threshold values should therefore be calibrated or adjusted for some purpose (18) in different cohorts. Secondly, subgroup analysis in the test set showed that the AUROC was highest in patients with ER+/HER2-, followed by patients with ER-/HER-, and lowest in patients with ER-/HER2+. The performance differences in the subgroups in the training set were consistent with those in the test set. These differences may suggest that the models are more accurate in the ER+/HER- patients than in other patients, but the ER+/HER- patients are not currently the main candidates for NAT. The different model performances in different subgroups may be due to the different intrinsic biological mechanisms of response to NAT in different BC subtypes, and the model cannot completely account for all subtypes. During model training, to get lower overall loss in gradient descent, the model may be optimized towards the subtype in which it was easier and more possible to make correct predictions, such as the ER+/HER2- subtype. This also suggests that in the future, when more training samples are collected, training different submodels in different subgroups and then integrating them may improve the overall performance and the robustness of the models in different subgroups. Lastly, and most importantly, the models should not be currently applied in clinical practice until they are validated in large prospective studies.

In summary, we developed the Ipredictor and ICpredictor models, which can accurately predict response to NAT across platforms in BC patients. The Ipredictor PS was also closely related to the immune and genomic mutation status. These results will contribute to individualized therapy for BC patients and the models are worthy of further validation in large prospective studies.

## Data availability statement

Publicly available datasets were analyzed in this study. This data can be found here: Gene Expression Omnibus (https://www.ncbi.nlm.nih.gov/geo/; accession number: GSE163882, GSE123845, and GSE20271), Sequence Read Archive (https://www.ncbi.nlm.nih.gov/sra; accession number: PRJNA688066), and The Cancer Genome Atlas (https://portal.gdc.cancer.gov/; accession number: TCGA-BRCA).

## Ethics statement

Ethical review and approval was not required for the study on human participants in accordance with the local legislation and institutional requirements. Written informed consent for participation was not required for this study in accordance with the national legislation and the institutional requirements.

## Author contributions

Conception and design: YP and XH. Data collection and cleaning: LH, XQ, and LL. Model development, validation, deployment, and exploration: JC. All authors contributed to the article and approved the submitted version.

## Funding

This work was supported by the Fundamental Research Funds for the Central Universities (WK9110000067) and the National Natural Science Foundation of China (82102905, 82172775).

## Acknowledgments

We thank the contributors and providers of GSE123845, GSE163882, GSE20271, and TCGA-BRCA for sharing the datasets in public databases.

## Conflict of interest

The authors declare that the research was conducted in the absence of any commercial or financial relationships that could be construed as a potential conflict of interest.

## Publisher’s note

All claims expressed in this article are solely those of the authors and do not necessarily represent those of their affiliated organizations, or those of the publisher, the editors and the reviewers. Any product that may be evaluated in this article, or claim that may be made by its manufacturer, is not guaranteed or endorsed by the publisher.
