# Supplementary material for: Machine learning models based on immunological genes to predict the response to neoadjuvant therapy in breast cancer patients
Source: Front Immunol. 2022 Jul 22;13:948601. doi: 10.3389/fimmu.2022.948601 (PMC9352856; doi:10.3389/fimmu.2022.948601)
Supplement: Supplementary file 1 [file Image_1.pdf]

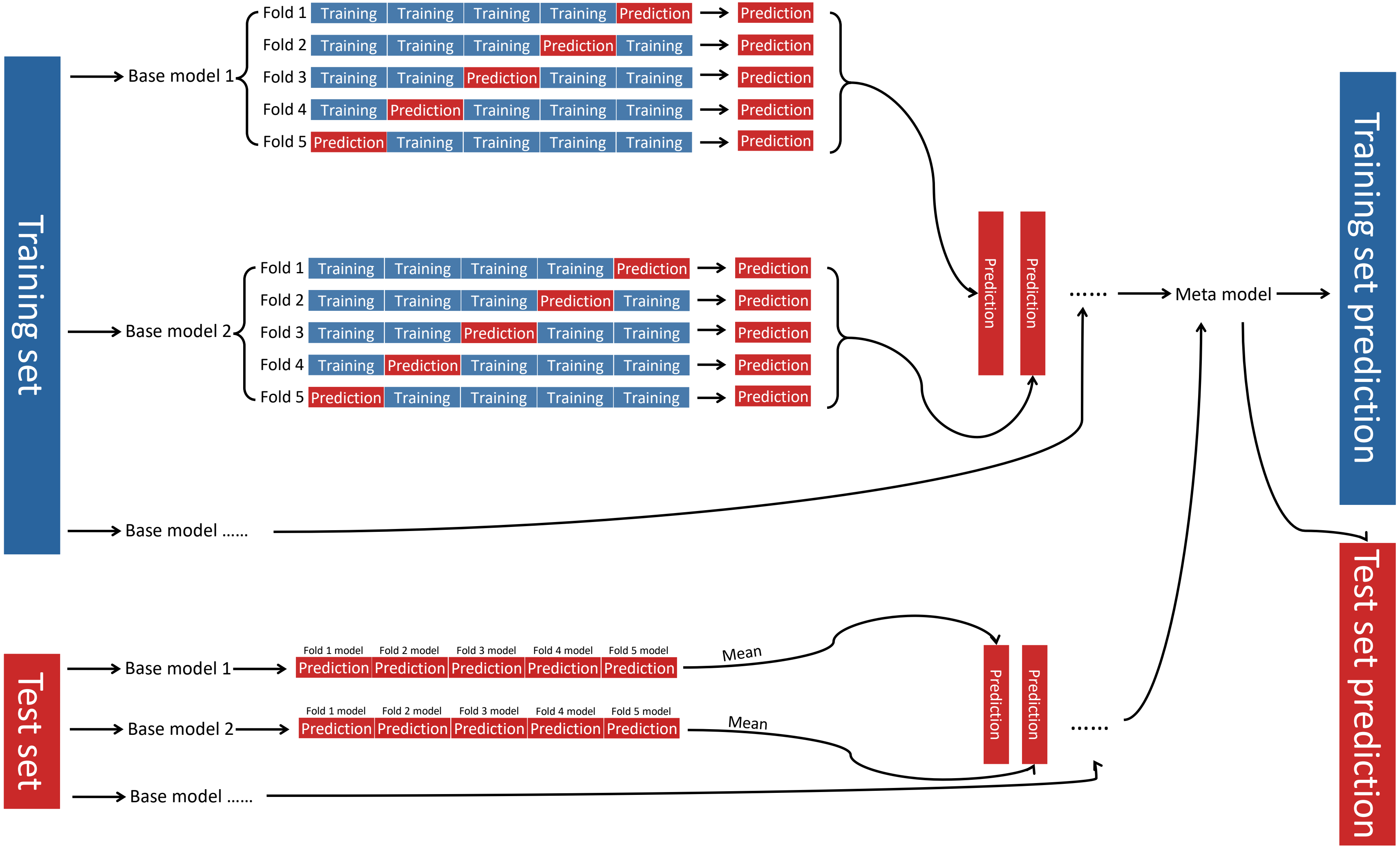

Supplementary Figure 1. Building stacking model. Each base model was trained on the four folds of the original training set and predicted the reserved training set. This process was repeated five times using different 4-fold subsets to generate the inputs of the meta models in the training set. Simultaneously, each base model also predicted the test set five times, and the mean of the predictions was the input of the meta models.
