# Supplementary material for: Machine learning models based on immunological genes to predict the response to neoadjuvant therapy in breast cancer patients
Source: Front Immunol. 2022 Jul 22;13:948601. doi: 10.3389/fimmu.2022.948601 (PMC9352856; doi:10.3389/fimmu.2022.948601)
Supplement: Supplementary file 2 [file Image_2.pdf]

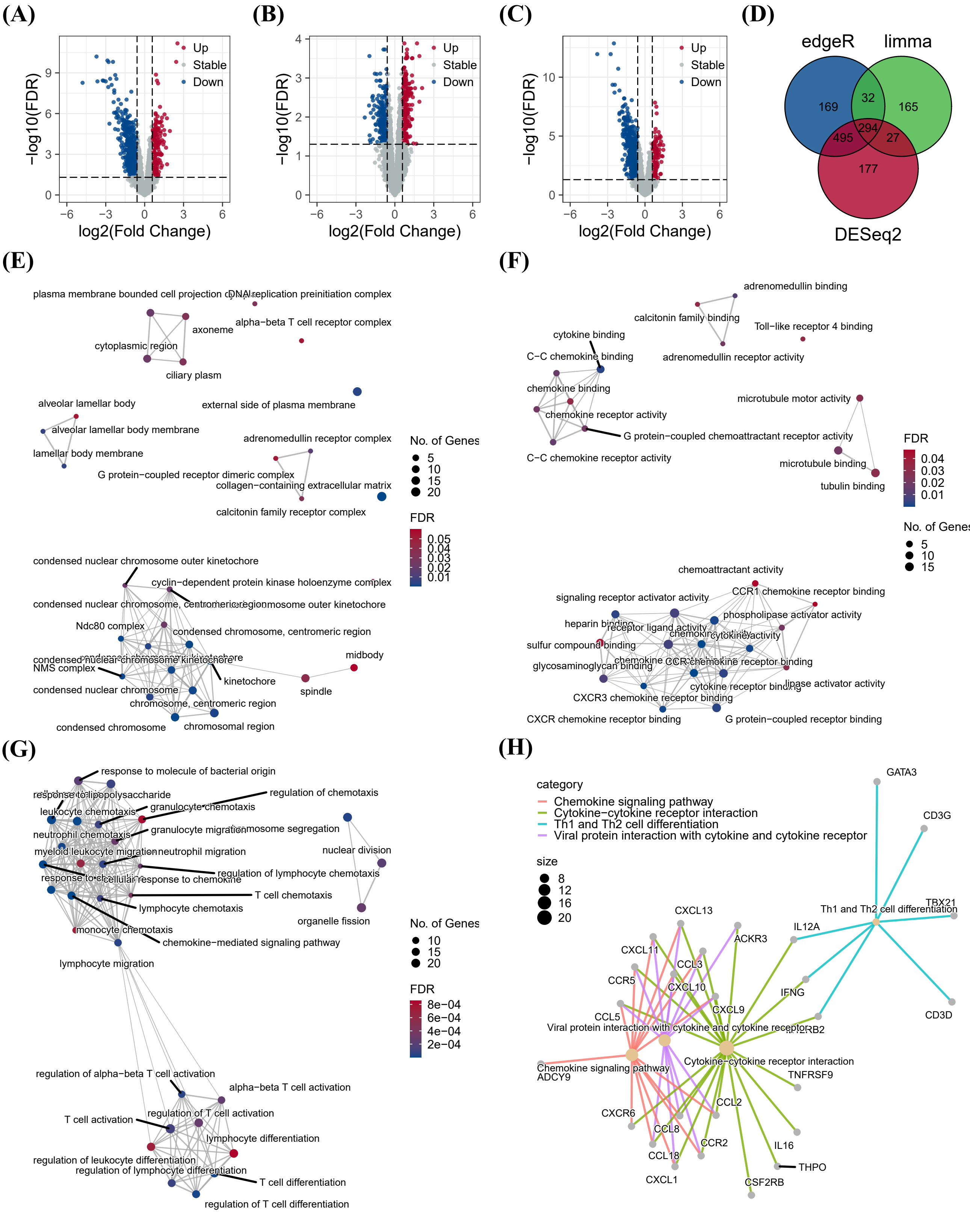

Supplementary Figure 2. Differential expression gene (DEG) analysis between patients who achieved pCR and those who did not by edgeR (A), DESeq2 (B), and limma (C). (D) overlapping DEGs. Over-representation analysis of DEGs for cellular component (E), molecular function (F), biological process (G), and KEGG pathways (H).
