# Supplementary material for: Machine learning models based on immunological genes to predict the response to neoadjuvant therapy in breast cancer patients
Source: Front Immunol. 2022 Jul 22;13:948601. doi: 10.3389/fimmu.2022.948601 (PMC9352856; doi:10.3389/fimmu.2022.948601)
Supplement: Supplementary file 3 [file Image_3.pdf]

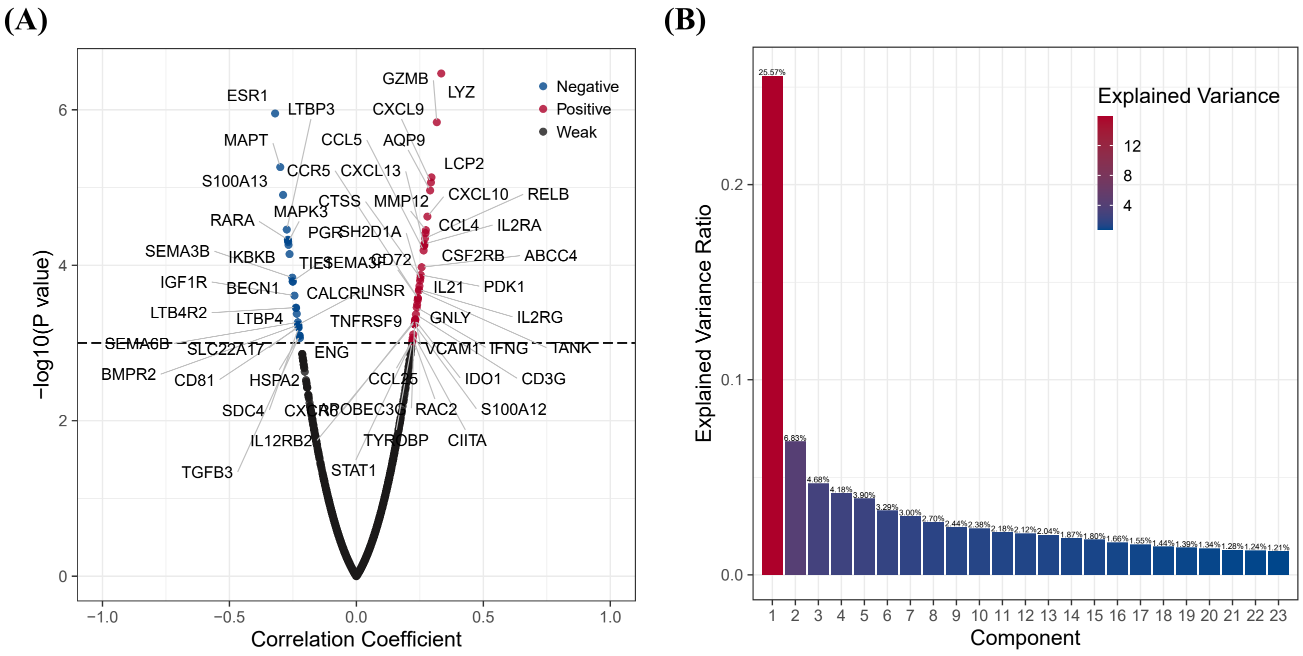

Supplementary Figure 3. Feature engineering. (A) Feature selection using Spearman rank correlation test. (B) Dimensionality reduction via PCA.
