# Supplementary material for: Machine learning models based on immunological genes to predict the response to neoadjuvant therapy in breast cancer patients
Source: Front Immunol. 2022 Jul 22;13:948601. doi: 10.3389/fimmu.2022.948601 (PMC9352856; doi:10.3389/fimmu.2022.948601)
Supplement: Supplementary file 6 [file Image_6.pdf]

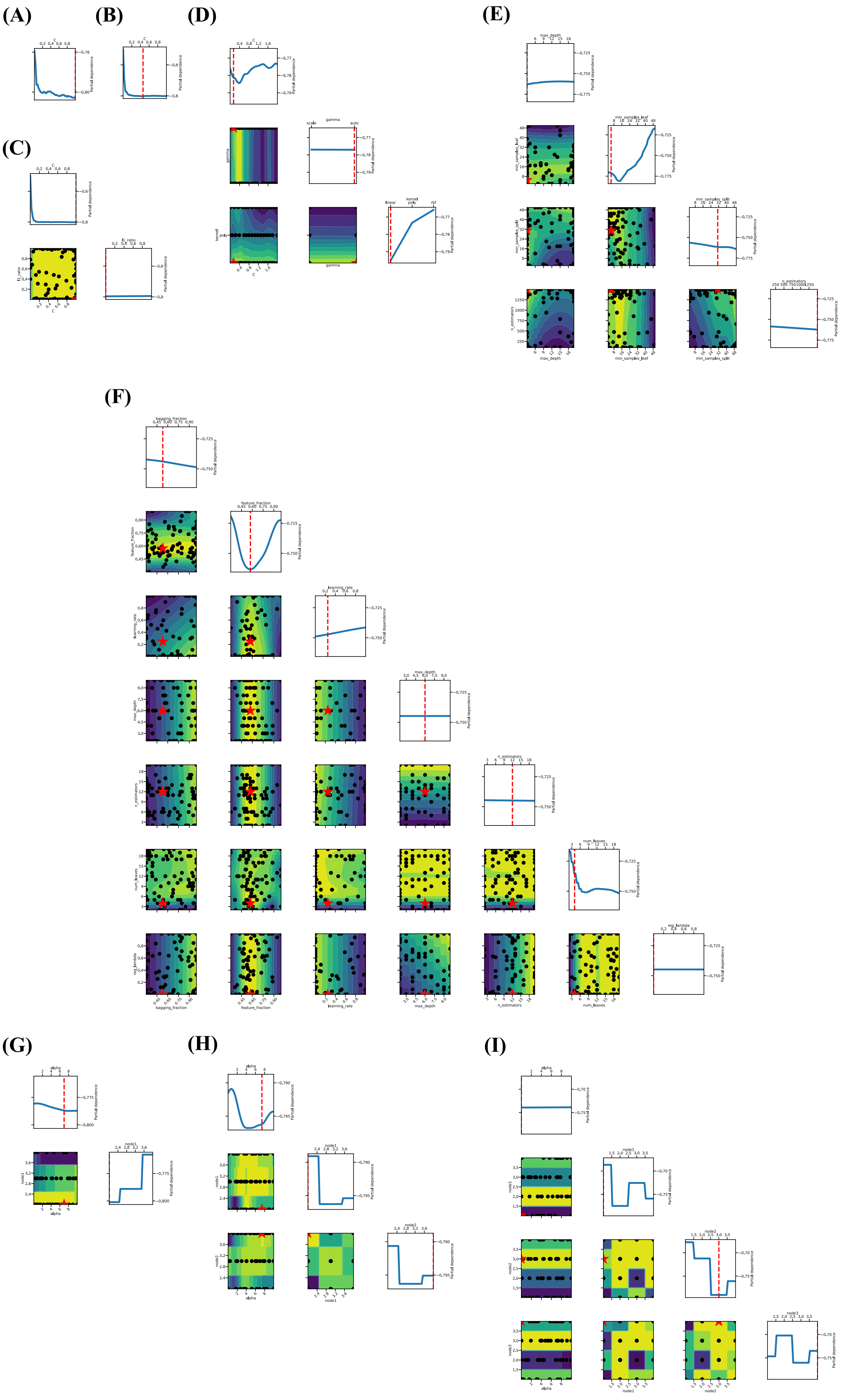

Supplementary Figure S6. Bayesian hyperparameter optimization for the candidate base models of the ICpredictor model. The plots on the diagonal lines show the effect of a single hyperparameter on the model performance, while the plots below the diagonal lines show the effects on the model performance when varying two hyperparameters. (A) Lasso. (B) RR. (C) ENR. (D) SVM. (E) RF. (F) lightGBM. (G) NNet1. (H) NNet2. (I) NNet3.
