# Supplementary material for: Machine learning models based on immunological genes to predict the response to neoadjuvant therapy in breast cancer patients
Source: Front Immunol. 2022 Jul 22;13:948601. doi: 10.3389/fimmu.2022.948601 (PMC9352856; doi:10.3389/fimmu.2022.948601)
Supplement: Supplementary file 7 [file Image_7.pdf]

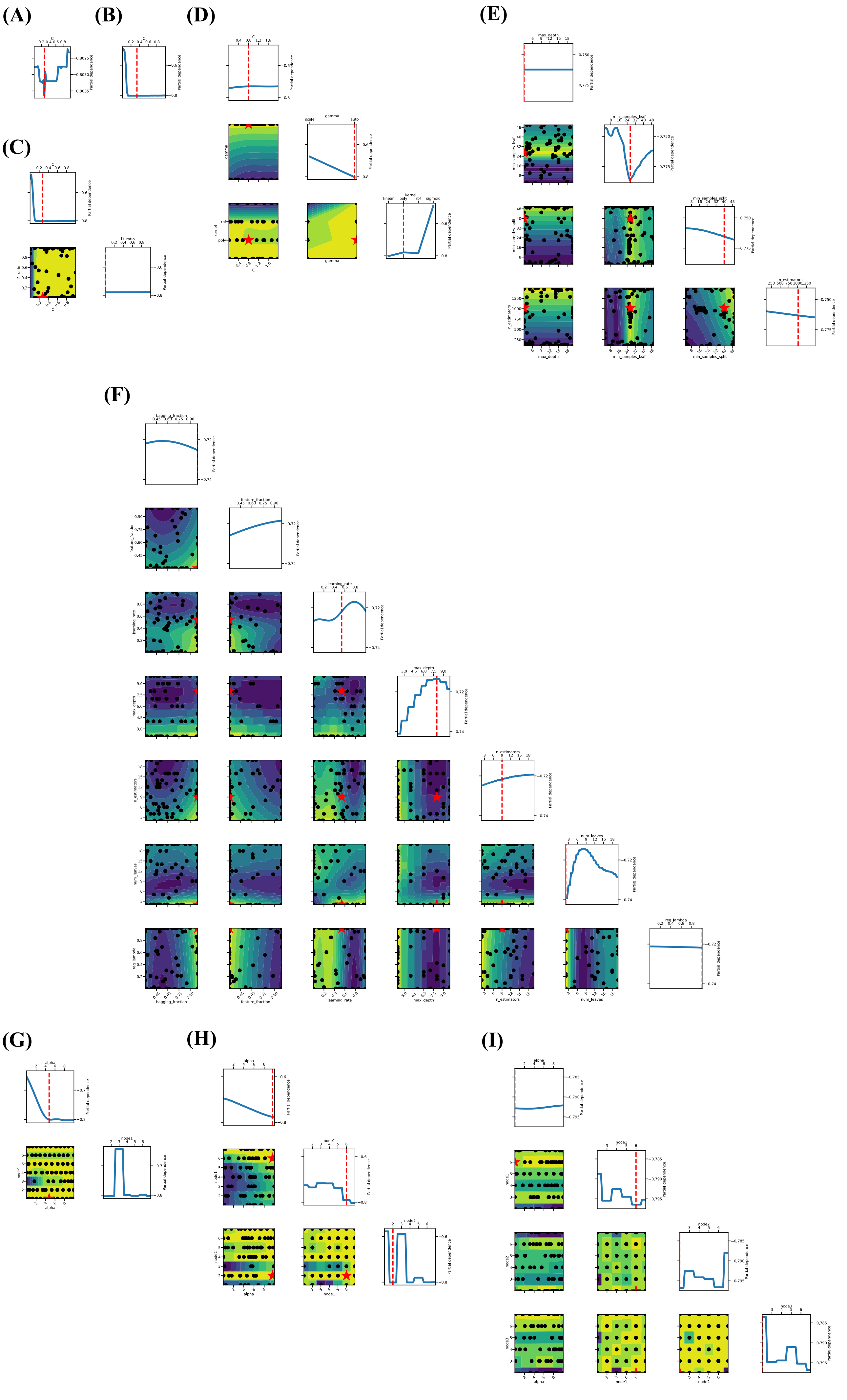

Supplementary Figure 7. Bayesian hyperparameter optimization for the candidate meta models of the ICpredictor model. The plots on the diagonal lines show the effect of a single hyperparameter on the model performance, while the plots below the diagonal lines show the effects on the model performance when varying two hyperparameters. (A) Lasso. (B) RR. (C) ENR. (D) SVM. (E) RF. (F) lightGBM. (G) NNet1. (H) NNet2. (I) NNet3.
