# Supplementary material for: Machine learning models based on immunological genes to predict the response to neoadjuvant therapy in breast cancer patients
Source: Front Immunol. 2022 Jul 22;13:948601. doi: 10.3389/fimmu.2022.948601 (PMC9352856; doi:10.3389/fimmu.2022.948601)
Supplement: Supplementary file 8 [file Image_8.pdf]

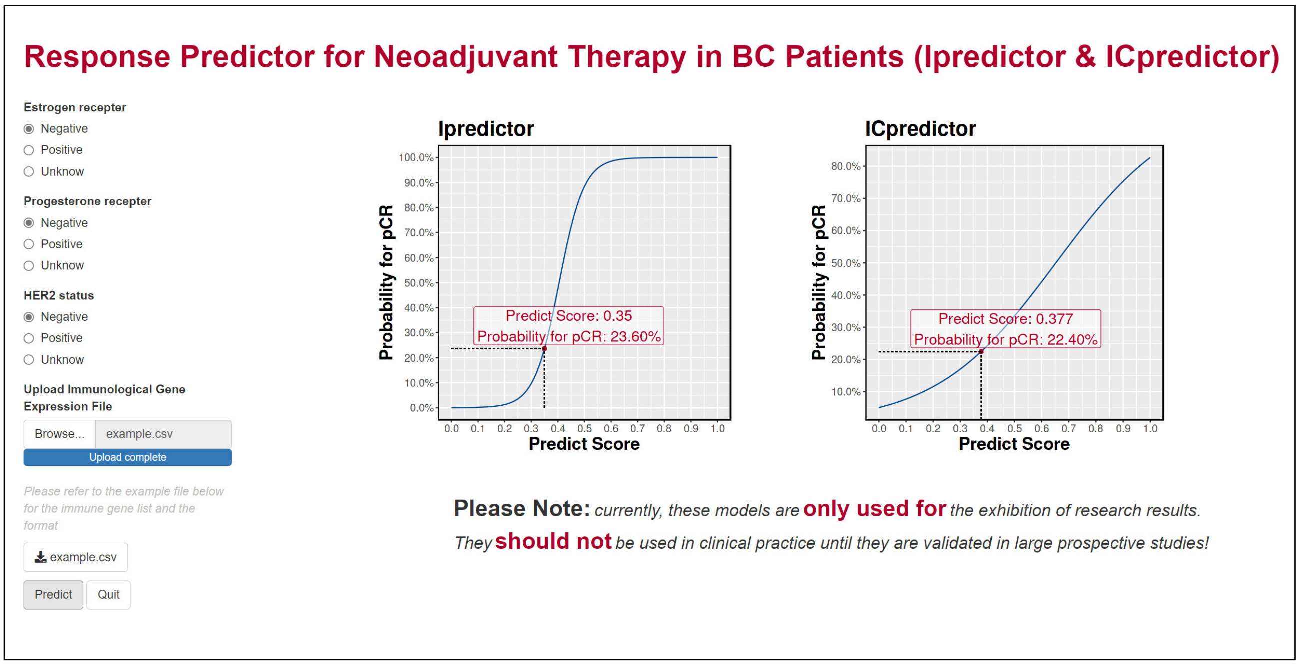

Supplementary Figure 8. A web-based application demonstration for the Ipredictor and ICpredictor models (available at https://www.jchen-lab.work:8082/imbc/). When ER status, PR status, HER2 status, and immunological gene expression file were inputted (left panel), it can calculate the predict scores and predict the pCR probabilities for the Ipredictor and ICpredictor models (right panel).
