# Supplementary material for: Machine learning models based on immunological genes to predict the response to neoadjuvant therapy in breast cancer patients
Source: Front Immunol. 2022 Jul 22;13:948601. doi: 10.3389/fimmu.2022.948601 (PMC9352856; doi:10.3389/fimmu.2022.948601)
Supplement: Supplementary file 9 [file Image_9.pdf]

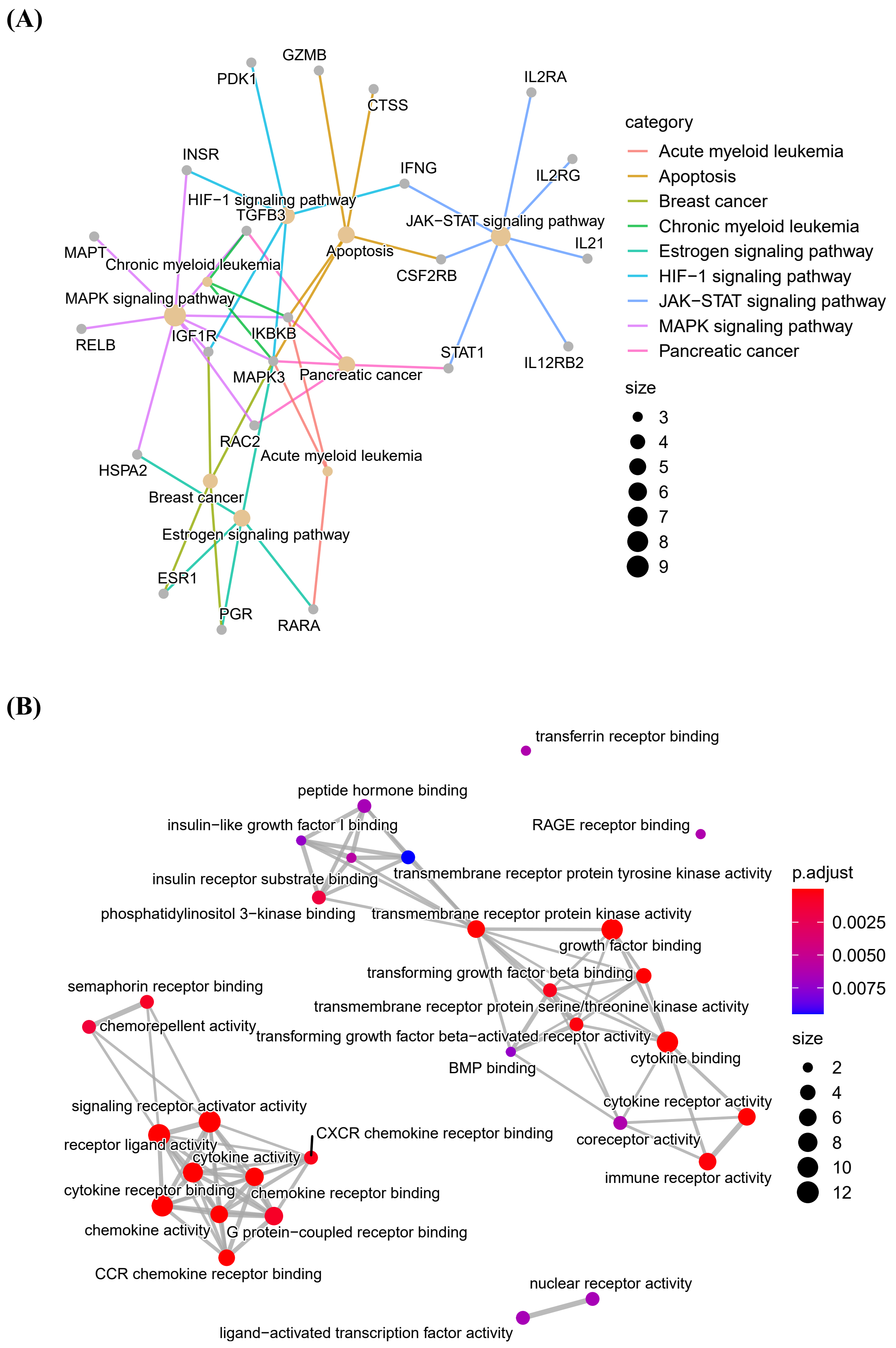

Supplementary Figure 9. KEGG pathway (A) and GO molecular function (B) over-representation analyses on the 62 pCR-related immunological genes.
