# Supplementary material for: Machine learning models based on immunological genes to predict the response to neoadjuvant therapy in breast cancer patients
Source: Front Immunol. 2022 Jul 22;13:948601. doi: 10.3389/fimmu.2022.948601 (PMC9352856; doi:10.3389/fimmu.2022.948601)
Supplement: Supplementary file 10 [file Image_10.pdf]

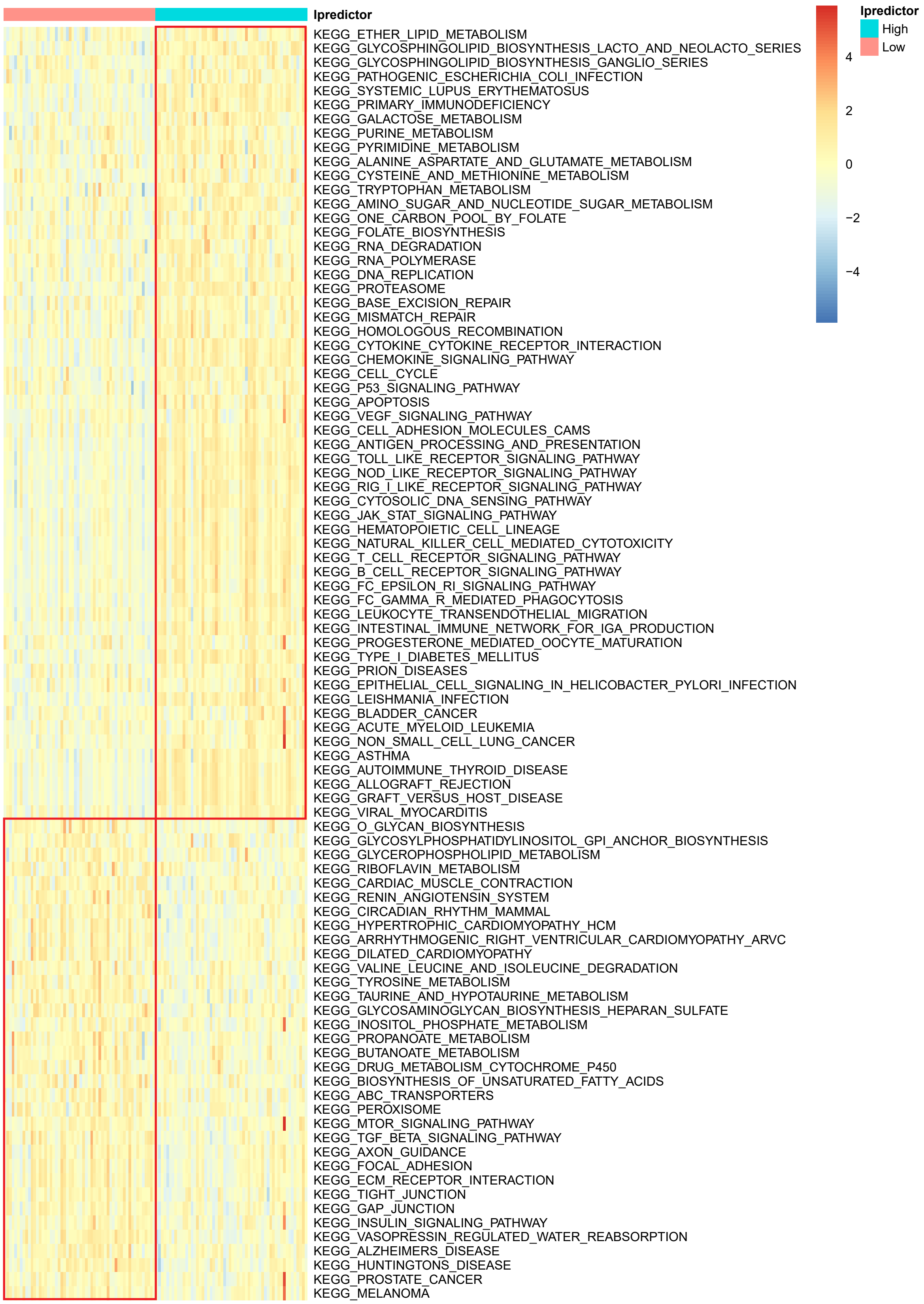

Supplementary Figure 10. Comparison of the KEGG pathway gene set enrichment scores between the patients with high and low PSs.
