# Supplementary material for: Machine learning models based on immunological genes to predict the response to neoadjuvant therapy in breast cancer patients
Source: Front Immunol. 2022 Jul 22;13:948601. doi: 10.3389/fimmu.2022.948601 (PMC9352856; doi:10.3389/fimmu.2022.948601)
Supplement: Supplementary file 12 [file Image_12.pdf]

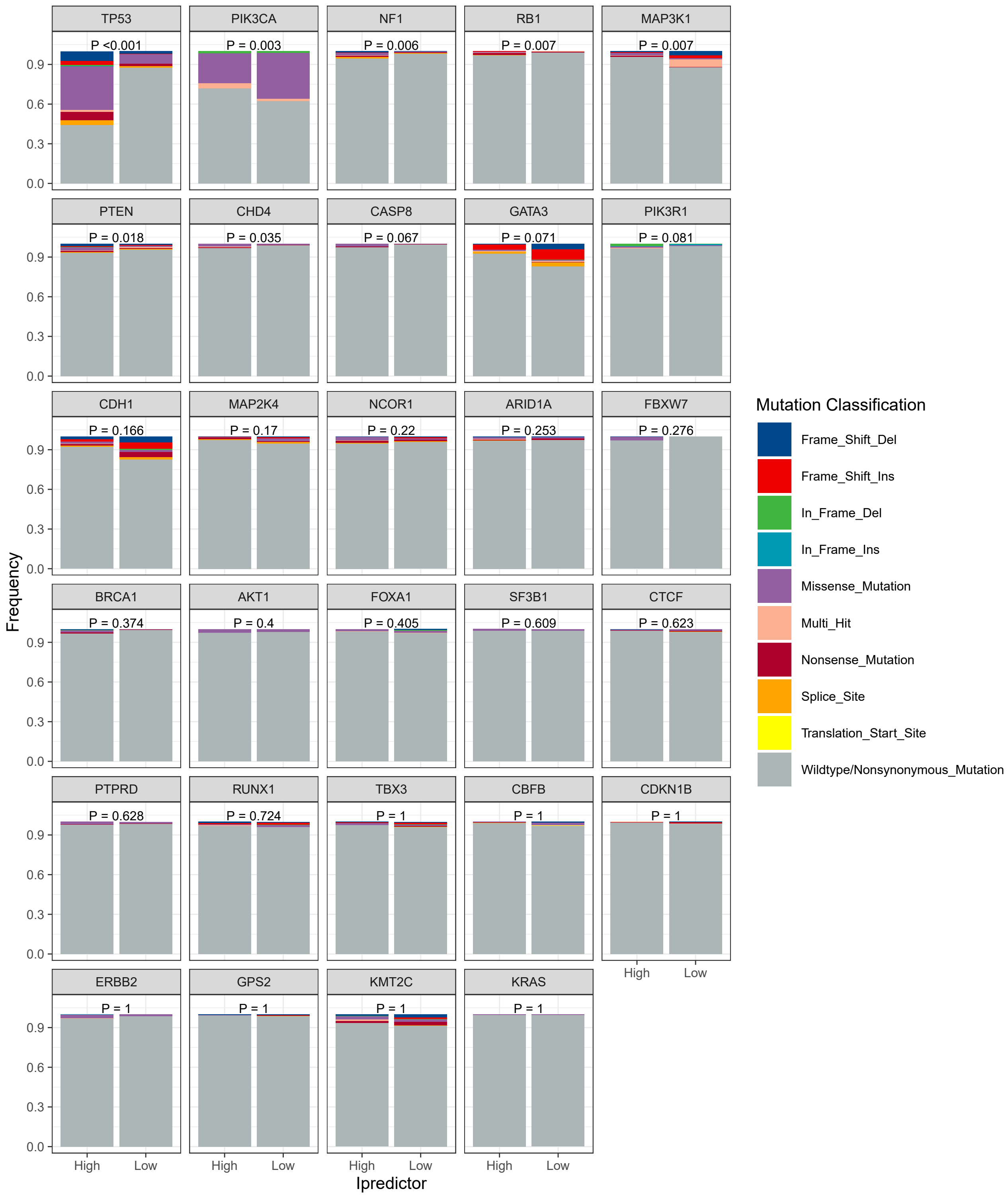

Supplementary Figure 12. Comparisons of BC driver gene mutation frequencies between patients with high and low PSs.
