# Supplementary material for: Machine learning models based on immunological genes to predict the response to neoadjuvant therapy in breast cancer patients
Source: Front Immunol. 2022 Jul 22;13:948601. doi: 10.3389/fimmu.2022.948601 (PMC9352856; doi:10.3389/fimmu.2022.948601)
Supplement: Supplementary file 13 [file Table_1.docx]

**Supplementary Table 1.** Clinicopathological characteristics of the training and test datasets

|  | **GSE163882 (n=222)** | **GSE123845 (n=112)** |
| --- | --- | --- |
| **Age** | 54.08±12.27 | 43.92±9.75 |
| **Menopausal Status (%)** |  |  |
| Post- | Not Available | 28 (25.0) |
| Pre- | Not Available | 83 (74.1) |
| Not Available | 222 (100.0) | 1 (0.9) |
| **ER Status (%)** |  |  |
| Positive | 105 (47.3) | 52 (46.4) |
| Negative | 117 (52.7) | 60 (53.6) |
| **PR Status (%)** |  |  |
| Positive | 72 (32.4) | 38 (33.9) |
| Negative | 150 (67.6) | 74 (66.1) |
| **HER2 Status (%)** |  |  |
| Negative | 159 (71.6) | 68 (60.7) |
| Positive | 63 (28.4) | 43 (38.4) |
| Not Available | 0 (0.0) | 1 (0.9) |
| **Ki67 status (%)** |  |  |
| High | Not Available | 77 (68.8) |
| Low | Not Available | 35 (31.2) |
| Not Available | 222 (100.0) | 0 (0.0) |
| **Histological Grade (%)** |  |  |
| 0 | 1 (0.5) | 0 (0.0) |
| 1 | 16 (7.2) | 11 (9.8) |
| 2 | 74 (33.3) | 34 (30.4) |
| 3 | 131 (59.0) | 37 (33.0) |
| Not Available | 0 (0.0) | 30 (26.8) |
| **Clinical T Stage (%)** |  |  |
| 1 | Not Available | 6 (5.4) |
| 2 | Not Available | 61 (54.5) |
| 3 | Not Available | 20 (17.9) |
| 4 | Not Available | 9 (8.0) |
| Not Available | 222 (100.0) | 16 (14.3) |
| **Clinical N Stage (%)** |  |  |
| 0 | Not Available | 3 (2.7) |
| 1 | Not Available | 20 (17.9) |
| 2 | Not Available | 17 (15.2) |
| 3 | Not Available | 24 (21.4) |
| Not Available | 222 (100.0) | 48 (42.9) |
| **Clinical Stage (%)** |  |  |
| 1 | 22 (9.9) | 0 (0.0) |
| 2 | 111 (50.0) | 24 (21.4) |
| 3 | 73 (32.9) | 50 (44.6) |
| 4 | 2 (0.9) | 1 (0.9) |
| Not Available | 14 (6.3) | 37 (33.0) |
| **Response (%)** |  |  |
| pCR | 80 (36.0) | 42 (37.5) |
| Non-pCR | 142 (64.0) | 70 (62.5) |
