# Supplementary material for: Machine learning models based on immunological genes to predict the response to neoadjuvant therapy in breast cancer patients
Source: Front Immunol. 2022 Jul 22;13:948601. doi: 10.3389/fimmu.2022.948601 (PMC9352856; doi:10.3389/fimmu.2022.948601)
Supplement: Supplementary file 14 [file Table_2.docx]

**Supplementary Table 2.** Software used in this study

| **Software** | **Version** | **Use** | **Source** |
| --- | --- | --- | --- |
| TrimGalore | 0.6.7 | Quality control and adapter trimming for RNA-seq data | https://www.bioinformatics.babraham.ac.uk/projects/trim_galore/ |
| HISAT2 | 2.2.1 | Alignment for RNA-seq data | https://daehwankimlab.github.io/hisat2/ |
| Samtools | 1.7 | Sort and index for aligned and mapped RNA-seq data | http://www.htslib.org/ |
| featureCounts | 2.0.1 | Counting reads for RNA-seq data | http://subread.sourceforge.net/ |
| edgeR | 3.32.1 | Identifying differential expression genes | http://bioinf.wehi.edu.au/edgeR |
| limma | 3.46.0 | Identifying differential expression genes | http://bioinf.wehi.edu.au/limma |
| DESeq2 | 1.30.1 | Identifying differential expression genes | https://github.com/mikelove/DESeq2 |
| clusterPorfiler | 3.18.1 | GO and KEGG over-representative analysis | https://bioconductor.org/packages/release/bioc/html/clusterProfiler.html |
| GSVA | 1.38.2 | Single-sample gene set enrichment analysis | https://github.com/rcastelo/GSVA |
| ImmuneSubtypeClassifier | 0.1.1 | Estimating the immune subtypes for GSE163882 | https://github.com/CRI-iAtlas/ImmuneSubtypeClassifier/ |
| estimate | 1.0.13 | Estimating the stromal and immune cell scores | https://bioinformatics.mdanderson.org/estimate/rpackage.html |
| CIBERSORTx | NA | Estimating the abundance of immune cell subgroups | https://cibersortx.stanford.edu/ |
| ModelMetrics | 1.2.2.2 | Calculating brier score, AUC, specificity, sensitivity, negative predictive value, and positive predictive value for the models | https://cran.r-project.org/web/packages/ModelMetrics/index.html |
| genefu | 2.22.1 | Calculating GGI, Oncotype DX scores, and MammaPrint scores | http://www.pmgenomics.ca/bhklab/software/genefu |
| boot | 1.3-27 | Bootstrap resampling | https://cran.r-project.org/web/packages/boot/index.html |
| qvalue | 2.22.0 | Estimate false discovery rate | http://github.com/jdstorey/qvalue |
| pheatmap | 1.0.12 | Plot heat map | https://cran.r-project.org/web/packages/pheatmap/index.html |
| dplyr | 1.0.7 | Data manipulation | https://www.tidyverse.org |
| ggsci | 2.9 | Color palettes | https://nanx.me/ggsci/ |
| ggplot2 | 3.3.5 | Data visualization | https://ggplot2.tidyverse.org |
| numpy | 1.21.2 | Scientific computing | https://numpy.org/ |
| pandas | 1.3.5 | Data manipulation | https://pandas.pydata.org/ |
| scipy | 1.7.3 | Scientific computing | https://scipy.org/ |
| scikit-learn | 1.0.2 | Model building | https://scikit-learn.org/ |
| lightgbm | 3.2.1 | Model building | https://www.microsoft.com/en-us/research/project/lightgbm/ |
| scikit-optimize | 0.9.0 | Bayesian optimization | https://scikit-optimize.github.io/ |
