# Supplementary material for: Machine learning models based on immunological genes to predict the response to neoadjuvant therapy in breast cancer patients
Source: Front Immunol. 2022 Jul 22;13:948601. doi: 10.3389/fimmu.2022.948601 (PMC9352856; doi:10.3389/fimmu.2022.948601)
Supplement: Supplementary file 15 [file Table_3.docx]

**Supplementary Table 3.** Bayesian hyperparameter optimization for the Ipredictor model

|  | **Model** | **Optimized Hyperparameter** | **Mean cv AUROC** |
| --- | --- | --- | --- |
| **Base model** | Lasso | C: 0.0707622255966804 | 0.748676108 |
|  | RR | C: 0.004550113982917889 | 0.741964286 |
|  | ENR | C: 0.06547099653362055 | 0.748245074 |
|  |  | l1_ratio: 0.9720749736643403 |  |
|  | SVM | C: 1e-05 | 0.736391626 |
|  |  | gamma: auto |  |
|  |  | kernel: linear |  |
|  | RF | max_depth: 3 | 0.708528325 |
|  |  | min_samples_leaf: 2 |  |
|  |  | min_samples_split: 12 |  |
|  |  | n_estimators: 888 |  |
|  | lightGBM | bagging_fraction: 0.7225545179057304 | 0.74171798 |
|  |  | feature_fraction: 0.5524457657747279 |  |
|  |  | learning_rate: 0.3789528070940167 |  |
|  |  | max_depth: 8.0 |  |
|  |  | n_estimators: 10.0 |  |
|  |  | num_leaves: 2.0 |  |
|  |  | reg_lambda: 0.001 |  |
|  | NNet1 | alpha: 5.914720167157277 | 0.717872537 |
|  |  | hidden_layer_sizes1: 2.0 |  |
|  | NNet2 | alpha: 10.0 | 0.731065271 |
|  |  | hidden_layer_sizes1: 7.0 |  |
|  |  | hidden_layer_sizes2: 2.0 |  |
|  | NNet3 | alpha: 8.44408032990687 | 0.743626847 |
|  |  | hidden_layer_sizes1: 5.0 |  |
|  |  | hidden_layer_sizes2: 23.0 |  |
|  |  | hidden_layer_sizes3: 2.0 |  |
| **Meta model** | Lasso | C: 0.7104869295015911 | 0.749538177 |
|  | RR | C: 0.04227704308818112 | 0.751000616 |
|  | ENR | C: 0.21168272577138167 | 0.750569581 |
|  |  | l1_ratio: 0.0001 |  |
|  | SVM | C: 2.0 | 0.751908867 |
|  |  | gamma: auto |  |
|  |  | kernel: poly |  |
|  | RF | max_depth: 4 | 0.743057266 |
|  |  | min_samples_leaf: 2 |  |
|  |  | min_samples_split: 50 |  |
|  |  | n_estimators: 100 |  |
|  | lightGBM | bagging_fraction: 0.30334733223506555 | 0.740863608 |
|  |  | feature_fraction: 0.3 |  |
|  |  | learning_rate: 0.23193951520173 |  |
|  |  | max_depth: 7.0 |  |
|  |  | n_estimators: 20.0 |  |
|  |  | num_leaves: 2.0 |  |
|  |  | reg_lambda: 0.912204105146965 |  |
|  | NNet1 | alpha: 1.6486710429965694 | 0.758589901 |
|  |  | hidden_layer_sizes1: 2.0 |  |
|  | NNet2 | alpha: 0.034452585963442146 | 0.755480296 |
|  |  | hidden_layer_sizes1: 2.0 |  |
|  |  | hidden_layer_sizes2: 1.0 |  |
|  | NNet3 | alpha: 2.8624864569016064 | 0.752386084 |
|  |  | hidden_layer_sizes1: 2.0 |  |
|  |  | hidden_layer_sizes2: 5.0 |  |
|  |  | hidden_layer_sizes3: 4.0 |  |
