# Supplementary material for: Machine learning models based on immunological genes to predict the response to neoadjuvant therapy in breast cancer patients
Source: Front Immunol. 2022 Jul 22;13:948601. doi: 10.3389/fimmu.2022.948601 (PMC9352856; doi:10.3389/fimmu.2022.948601)
Supplement: Supplementary file 16 [file Table_4.docx]

**Supplementary Table 4.** Bayesian hyperparameter optimization for the ICpredictor model

|  | **Model** | **Optimized Hyperparameter** | **Mean cv AUROC** |
| --- | --- | --- | --- |
| **Base model** | Lasso | C: 0.4602338728414804 | 0.802309113 |
|  | RR | C: 1.0 | 0.80278633 |
|  | ENR | C: 1.0 | 0.80278633 |
|  |  | l1_ratio: 0.0001 |  |
|  | SVM | C: 0.14361185501623822 | 0.801524015 |
|  |  | gamma: auto |  |
|  |  | kernel: linear |  |
|  | RF | max_depth: 3 | 0.793026478 |
|  |  | min_samples_leaf: 5 |  |
|  |  | min_samples_split: 31 |  |
|  |  | n_estimators: 1500 |  |
|  | lightGBM | bagging_fraction: 0.5314578487617004 | 0.781011392 |
|  |  | feature_fraction: 0.5734586152706567 |  |
|  |  | learning_rate: 0.24815881626208858 |  |
|  |  | max_depth: 6.0 |  |
|  |  | n_estimators: 12.0 |  |
|  |  | num_leaves: 4.0 |  |
|  |  | reg_lambda: 0.001 |  |
|  | NNet1 | alpha: 6.972544381270246 | 0.80278633 |
|  |  | hidden_layer_sizes1: 2.0 |  |
|  | NNet2 | alpha: 7.4169131972541305 | 0.807219828 |
|  |  | hidden_layer_sizes1: 2.0 |  |
|  |  | hidden_layer_sizes2: 4.0 |  |
|  | NNet3 | alpha: 1e-05 | 0.804618227 |
|  |  | hidden_layer_sizes1: 1.0 |  |
|  |  | hidden_layer_sizes2: 3.0 |  |
|  |  | hidden_layer_sizes3: 4.0 |  |
| **Meta model** | Lasso | C: 0.3382870055848667 | 0.80278633 |
|  | RR | C: 0.2814831150756039 | 0.803648399 |
|  | ENR | C: 0.27072484885817666 | 0.803648399 |
|  |  | l1_ratio: 0.0001 |  |
|  | SVM | C: 0.8046821560696932 | 0.802770936 |
|  |  | gamma: auto |  |
|  |  | kernel: poly |  |
|  | RF | max_depth: 3 | 0.787815579 |
|  |  | min_samples_leaf: 27 |  |
|  |  | min_samples_split: 40 |  |
|  |  | n_estimators: 1014 |  |
|  | lightGBM | bagging_fraction: 1.0 | 0.783181958 |
|  |  | feature_fraction: 0.3 |  |
|  |  | learning_rate: 0.5391952567804994 |  |
|  |  | max_depth: 8.0 |  |
|  |  | n_estimators: 9.0 |  |
|  |  | num_leaves: 2.0 |  |
|  |  | reg_lambda: 1.0 |  |
|  | NNet1 | alpha: 4.75710038287535 | 0.80455665 |
|  |  | hidden_layer_sizes1: 1.0 |  |
|  | NNet2 | alpha: 9.636662160715813 | 0.804094828 |
|  |  | hidden_layer_sizes1: 6.0 |  |
|  |  | hidden_layer_sizes2: 2.0 |  |
|  | NNet3 | alpha: 1e-05 | 0.803679187 |
|  |  | hidden_layer_sizes1: 6.0 |  |
|  |  | hidden_layer_sizes2: 2.0 |  |
|  |  | hidden_layer_sizes3: 2.0 |  |
