# Supplementary material for: Machine learning models based on immunological genes to predict the response to neoadjuvant therapy in breast cancer patients
Source: Front Immunol. 2022 Jul 22;13:948601. doi: 10.3389/fimmu.2022.948601 (PMC9352856; doi:10.3389/fimmu.2022.948601)
Supplement: Supplementary file 17 [file Table_5.docx]

**Supplementary Table 5.** Brier scores of the Ipredictor model, ICpredictor model, and clinicopathological characteristics in the training and test datasets

|  |  | **Brier Score** | **Standard Deviation** | **95% Confidence Interval** |
| --- | --- | --- | --- | --- |
| **Training set** | Ipredictor | 0.189 | 0.012 | 0.167-0.213 |
|  | ICpredictor | 0.174 | 0.012 | 0.15-0.198 |
|  | Age | 0.224 | 0.005 | 0.213-0.234 |
|  | ER Status | 0.215 | 0.008 | 0.2-0.231 |
|  | PR Status | 0.207 | 0.008 | 0.191-0.223 |
|  | HER2 Status | 0.227 | 0.004 | 0.218-0.235 |
|  | Histological Grade | 0.216 | 0.007 | 0.203-0.229 |
|  | Clinical Stage | 0.226 | 0.004 | 0.218-0.234 |
|  | ER/PR/HER2 | 0.219 | 0.007 | 0.207-0.233 |
|  | CPpredictor | 0.179 | 0.012 | 0.155-0.202 |
| **Test set** | Ipredictor | 0.194 | 0.015 | 0.163-0.224 |
|  | ICpredictor | 0.187 | 0.016 | 0.157-0.22 |
|  | Age | 0.234 | 0.002 | 0.231-0.237 |
|  | Menopausal Status | 0.227 | 0.007 | 0.213-0.241 |
|  | ER Status | 0.228 | 0.007 | 0.214-0.243 |
|  | PR Status | 0.22 | 0.01 | 0.203-0.241 |
|  | HER2 Status | 0.228 | 0.008 | 0.212-0.243 |
|  | Ki67 Status | 0.234 | 0.004 | 0.227-0.241 |
|  | ER/PR/HER2 | 0.229 | 0.007 | 0.214-0.243 |
|  | Histological Grade | 0.225 | 0.008 | 0.21-0.242 |
|  | Clinical T stage | 0.207 | 0.012 | 0.184-0.232 |
|  | Clinical N stage | 0.201 | 0.014 | 0.173-0.229 |
|  | Clinical stage | 0.209 | 0.017 | 0.178-0.243 |
|  | CPpredictor | 0.202 | 0.017 | 0.171-0.235 |
