# Supplementary material for: Machine learning models based on immunological genes to predict the response to neoadjuvant therapy in breast cancer patients
Source: Front Immunol. 2022 Jul 22;13:948601. doi: 10.3389/fimmu.2022.948601 (PMC9352856; doi:10.3389/fimmu.2022.948601)
Supplement: Supplementary file 18 [file Table_6.docx]

**Supplementary Table 6.** AUPRCs of the Ipredictor model, ICpredictor model, and clinicopathological characteristics in the training and test datasets

|  |  | **AUPRC** | **Standard Deviation** | **95% Confidence Interval** |
| --- | --- | --- | --- | --- |
| **Training set** | Ipredictor | 0.636 | 0.051 | 0.541-0.737 |
|  | ICpredictor | 0.702 | 0.045 | 0.614-0.787 |
|  | Age | 0.463 | 0.044 | 0.383-0.556 |
|  | ER Status | 0.457 | 0.027 | 0.405-0.514 |
|  | PR Status | 0.459 | 0.021 | 0.422-0.503 |
|  | HER2 Status | 0.416 | 0.032 | 0.365-0.487 |
|  | Histological Grade | 0.446 | 0.024 | 0.401-0.493 |
|  | Clinical Stage | 0.393 | 0.03 | 0.344-0.461 |
|  | ER/PR/HER2 | 0.429 | 0.029 | 0.376-0.489 |
|  | CPpredictor | 0.657 | 0.049 | 0.564-0.753 |
| **Test set** | Ipredictor | 0.64 | 0.063 | 0.518-0.764 |
|  | ICpredictor | 0.658 | 0.064 | 0.532-0.787 |
|  | Age | 0.431 | 0.05 | 0.376-0.561 |
|  | Menopausal Status | 0.438 | 0.046 | 0.372-0.546 |
|  | ER Status | 0.434 | 0.034 | 0.381-0.512 |
|  | PR Status | 0.45 | 0.031 | 0.395-0.513 |
|  | HER2 Status | 0.452 | 0.042 | 0.385-0.548 |
|  | Ki67 Status | 0.418 | 0.041 | 0.365-0.519 |
|  | ER/PR/HER2 | 0.423 | 0.039 | 0.361-0.511 |
|  | Histological Grade | 0.475 | 0.036 | 0.411-0.554 |
|  | Clinical T stage | 0.53 | 0.054 | 0.432-0.636 |
|  | Clinical N stage | 0.58 | 0.057 | 0.473-0.686 |
|  | Clinical stage | 0.537 | 0.06 | 0.431-0.659 |
|  | CPpredictor | 0.55 | 0.077 | 0.438-0.734 |
