# Supplementary material for: Machine learning models based on immunological genes to predict the response to neoadjuvant therapy in breast cancer patients
Source: Front Immunol. 2022 Jul 22;13:948601. doi: 10.3389/fimmu.2022.948601 (PMC9352856; doi:10.3389/fimmu.2022.948601)
Supplement: Supplementary file 19 [file Table_7.docx]

**Supplementary Table 7.** AUROCs of the Ipredictor model, ICpredictor model, and clinicopathological characteristics in the training and test datasets

|  |  | **AUROC** | **Standard Deviation** | **95% Confidence Interval** |
| --- | --- | --- | --- | --- |
| **Training set** | Ipredictor | 0.749 | 0.035 | 0.678-0.813 |
|  | ICpredictor | 0.801 | 0.03 | 0.743-0.856 |
|  | Age | 0.593 | 0.039 | 0.515-0.668 |
|  | ER Status | 0.635 | 0.033 | 0.568-0.699 |
|  | PR Status | 0.656 | 0.028 | 0.603-0.711 |
|  | HER2 Status | 0.562 | 0.032 | 0.506-0.628 |
|  | Histological Grade | 0.632 | 0.032 | 0.568-0.691 |
|  | Clinical Stage | 0.575 | 0.034 | 0.509-0.642 |
|  | ER/PR/HER2 | 0.622 | 0.036 | 0.549-0.688 |
|  | CPpredictor | 0.782 | 0.031 | 0.721-0.844 |
| **Test set** | Ipredictor | 0.745 | 0.045 | 0.652-0.829 |
|  | ICpredictor | 0.769 | 0.043 | 0.68-0.846 |
|  | Age | 0.503 | 0.035 | 0.502-0.635 |
|  | Menopausal Status | 0.571 | 0.041 | 0.504-0.657 |
|  | ER Status | 0.586 | 0.045 | 0.51-0.679 |
|  | PR Status | 0.619 | 0.043 | 0.533-0.7 |
|  | HER2 Status | 0.591 | 0.045 | 0.509-0.684 |
|  | Ki67 Status | 0.544 | 0.041 | 0.502-0.646 |
|  | ER/PR/HER2 | 0.595 | 0.049 | 0.509-0.694 |
|  | Histological Grade | 0.629 | 0.048 | 0.53-0.721 |
|  | Clinical T stage | 0.678 | 0.042 | 0.594-0.757 |
|  | Clinical N stage | 0.629 | 0.036 | 0.559-0.7 |
|  | Clinical stage | 0.664 | 0.052 | 0.562-0.761 |
|  | CPpredictor | 0.731 | 0.055 | 0.621-0.833 |
