# Supplementary material for: Machine learning models based on immunological genes to predict the response to neoadjuvant therapy in breast cancer patients
Source: Front Immunol. 2022 Jul 22;13:948601. doi: 10.3389/fimmu.2022.948601 (PMC9352856; doi:10.3389/fimmu.2022.948601)
Supplement: Supplementary file 20 [file Table_8.docx]

**Supplementary Table 8.** SENs, SPEs, NPVs, and PPVs of the Ipredictor and ICpredictor models in the training and test sets

|  |  |  | **Origin** | **Standard Deviation** | **95% Confidence Interval** |
| --- | --- | --- | --- | --- | --- |
| **Training set** | **Ipredictor** | SPE | 0.739 | 0.037 | 0.669-0.81 |
|  |  | SEN | 0.637 | 0.054 | 0.537-0.738 |
|  |  | NPV | 0.784 | 0.027 | 0.734-0.836 |
|  |  | PPV | 0.58 | 0.039 | 0.505-0.655 |
|  | **ICpredictor** | SPE | 0.683 | 0.038 | 0.613-0.761 |
|  |  | SEN | 0.787 | 0.045 | 0.7-0.875 |
|  |  | NPV | 0.851 | 0.028 | 0.795-0.904 |
|  |  | PPV | 0.583 | 0.033 | 0.52-0.653 |
| **Test set** | **Ipredictor** | SPE | 0.957 | 0.024 | 0.9-1 |
|  |  | SEN | 0.31 | 0.07 | 0.167-0.452 |
|  |  | NPV | 0.698 | 0.022 | 0.657-0.742 |
|  |  | PPV | 0.812 | 0.094 | 0.615-1 |
|  | **ICpredictor** | SPE | 0.841 | 0.044 | 0.754-0.928 |
|  |  | SEN | 0.5 | 0.079 | 0.357-0.643 |
|  |  | NPV | 0.734 | 0.032 | 0.675-0.8 |
|  |  | PPV | 0.656 | 0.072 | 0.525-0.794 |
