# Supplementary material for: Machine learning models based on immunological genes to predict the response to neoadjuvant therapy in breast cancer patients
Source: Front Immunol. 2022 Jul 22;13:948601. doi: 10.3389/fimmu.2022.948601 (PMC9352856; doi:10.3389/fimmu.2022.948601)
Supplement: Supplementary file 21 [file Table_9.docx]

**Supplementary Table 9.** Univariate and multivariate analysis

|  |  |  | **Univariate Analysis** | | |  | **Multivariate Analysis** ^a^ | | | | | | |
| --- | --- | --- | --- | --- | --- | --- | --- | --- | --- | --- | --- | --- | --- |
|  |  |  | **Odds Ratio** | **95% Confidence Interval** | **P value** |  | **Odds Ratio** | **95% Confidence Interval** | **P value** |  | **Odds Ratio** | **95% Confidence Interval** | **P value** |
| **Training set** | Age ^b^ | ≤54 yrs |  |  |  |  |  |  |  |  |  |  |  |
|  |  | >54 yrs | 0.534 | 0.304-0.929 | 0.028 |  | 0.702 | 0.347-1.41 | 0.321 |  | 0.675 | 0.329-1.372 | 0.279 |
|  | ER | negative |  |  |  |  |  |  |  |  |  |  |  |
|  |  | positive | 0.323 | 0.178-0.572 | <0.001 |  | 2.537 | 0.962-7.131 | 0.066 |  | 1.902 | 0.726-5.275 | 0.2 |
|  | PR | negative |  |  |  |  |  |  |  |  |  |  |  |
|  |  | positive | 0.184 | 0.084-0.373 | <0.001 |  | 0.69 | 0.165-3.245 | 0.619 |  | 1.048 | 0.239-5.243 | 0.952 |
|  | HER2 | negative |  |  |  |  |  |  |  |  |  |  |  |
|  |  | positive | 1.806 | 0.992-3.286 | 0.052 |  |  |  |  |  |  |  |  |
|  | Grade | 1 |  |  |  |  |  |  |  |  |  |  |  |
|  |  | 2 | 2.25 | 0.556-15.198 | 0.313 |  | 0.836 | 0.16-6.633 | 0.845 |  | 0.991 | 0.175-8.787 | 0.993 |
|  |  | 3 | 5.915 | 1.57-38.634 | 0.022 |  | 0.795 | 0.139-6.534 | 0.808 |  | 1.169 | 0.199-10.373 | 0.873 |
|  | Stage | 1 |  |  |  |  |  |  |  |  |  |  |  |
|  |  | 2 | 1.285 | 0.508-3.449 | 0.603 |  |  |  |  |  |  |  |  |
|  |  | 3/4 | 0.594 | 0.218-1.686 | 0.313 |  |  |  |  |  |  |  |  |
|  | CPpredictor ^b^ | High |  |  |  |  |  |  |  |  |  |  |  |
|  |  | Low | 0.106 | 0.046-0.22 | <0.001 |  | 0.106 | 0.023-0.396 | 0.002 |  | 0.15 | 0.034-0.559 | 0.007 |
|  | Ipredictor ^b^ | High |  |  |  |  |  |  |  |  |  |  |  |
|  |  | Low | 0.204 | 0.112-0.364 | <0.001 |  | 0.288 | 0.133-0.601 | 0.001 |  |  |  |  |
|  | ICpredictor ^b^ | High |  |  |  |  |  |  |  |  |  |  |  |
|  |  | Low | 0.125 | 0.064-0.233 | <0.001 |  |  |  |  |  | 0.215 | 0.098-0.452 | <0.001 |
| **Test set** | Age ^b^ | ≤44 yrs |  |  |  |  |  |  |  |  |  |  |  |
|  |  | >44 yrs | 1 | 0.464-2.155 | 1 |  |  |  |  |  |  |  |  |
|  | Menopausal | pre- |  |  |  |  |  |  |  |  |  |  |  |
|  |  | post- | 2.074 | 0.866-5.003 | 0.101 |  |  |  |  |  |  |  |  |
|  | ER | negative |  |  |  |  |  |  |  |  |  |  |  |
|  |  | positive | 0.495 | 0.222-1.077 | 0.08 |  |  |  |  |  |  |  |  |
|  | PR | negative |  |  |  |  |  |  |  |  |  |  |  |
|  |  | positive | 0.314 | 0.12-0.748 | 0.012 |  | 4.694 | 0.208-162.355 | 0.336 |  | 5.849 | 0.339-182.652 | 0.238 |
|  | HER2 | negative |  |  |  |  |  |  |  |  |  |  |  |
|  |  | positive | 2.136 | 0.974-4.746 | 0.059 |  |  |  |  |  |  |  |  |
|  | Ki67 | High |  |  |  |  |  |  |  |  |  |  |  |
|  |  | Low | 0.978 | 0.421-2.22 | 0.958 |  |  |  |  |  |  |  |  |
|  | Grade | 1 |  |  |  |  |  |  |  |  |  |  |  |
|  |  | 2 | 0.097 | 0.016-0.467 | 0.005 |  | 0.094 | 0.007-0.814 | 0.042 |  | 0.054 | 0.004-0.468 | 0.013 |
|  |  | 3 | 1 | 0.285-3.506 | 1 |  | 0.624 | 0.095-3.65 | 0.605 |  | 0.597 | 0.094-3.572 | 0.572 |
|  | T stage | 1 |  |  |  |  |  |  |  |  |  |  |  |
|  |  | 2 | 0.333 | 0.045-1.668 | 0.208 |  | 0 | NA | 0.991 |  | 0 | NA | 0.993 |
|  |  | 3/4 | 0.088 | 0.011-0.493 | 0.009 |  | 0 | NA | 0.99 |  | 0 | NA | 0.993 |
|  | N stage | 0 |  |  |  |  |  |  |  |  |  |  |  |
|  |  | 1-3 | 0.074 | 0.011-0.291 | 0.001 |  | 0.063 | 0.004-0.539 | 0.023 |  | 0.076 | 0.005-0.641 | 0.031 |
|  | Stage | 2 |  |  |  |  |  |  |  |  |  |  |  |
|  |  | 3/4 | 0.237 | 0.088-0.609 | 0.003 |  | 0.817 | 0.159-4.288 | 0.807 |  | 0.604 | 0.106-3.249 | 0.556 |
|  | CPpredictor | High |  |  |  |  |  |  |  |  |  |  |  |
|  |  | Low | 0.291 | 0.095-0.789 | 0.02 |  | 0.199 | 0.005-4.636 | 0.324 |  | 0.241 | 0.008-4.242 | 0.346 |
|  | Ipredictor ^b^ | High |  |  |  |  |  |  |  |  |  |  |  |
|  |  | Low | 0.1 | 0.022-0.337 | <0.001 |  | 0.162 | 0.037-0.572 | 0.008 |  |  |  |  |
|  | ICpredictor ^b^ | High |  |  |  |  |  |  |  |  |  |  |  |
|  |  | Low | 0.19 | 0.076-0.45 | <0.001 |  |  |  |  |  | 0.105 | 0.021-0.42 | 0.003 |

^a^ Because the Ipredictor PS and ICpredictor PS were significantly correlated (r = 0.861 and r = 0.775 in the training and test set respectively), multivariate analysis was performed by combining clinicopathological characteristics with the Ipredictor PS and ICpredictor PS respectively to avoid multicollinearity.

^b^ Grouped according to the median or optimal threshold value.
