# Supplementary material for: Machine learning models based on immunological genes to predict the response to neoadjuvant therapy in breast cancer patients
Source: Front Immunol. 2022 Jul 22;13:948601. doi: 10.3389/fimmu.2022.948601 (PMC9352856; doi:10.3389/fimmu.2022.948601)
Supplement: Supplementary file 22 [file Table_10.docx]

**Supplementary Table 10.** Subgroup analysis

|  |  | **Subgroup** | **AUROC** | **Standard Deviation** | **95% Confidence Interval** |
| --- | --- | --- | --- | --- | --- |
| **Training set** | **Ipredictor** | **Age** |  |  |  |
|  |  | >54 yrs | 0.807 | 0.024 | 0.8-0.84 |
|  |  | ≤54 yrs | 0.698 | 0.073 | 0.586-0.723 |
|  |  | **ER** |  |  |  |
|  |  | negative | 0.725 | 0.049 | 0.672-0.766 |
|  |  | positive | 0.698 | 0.034 | 0.653-0.713 |
|  |  | **PR** |  |  |  |
|  |  | negative | 0.725 | 0.038 | 0.682-0.744 |
|  |  | positive | 0.619 | 0.023 | 0.479-0.522 |
|  |  | **HER2** |  |  |  |
|  |  | negative | 0.798 | 0.038 | 0.776-0.839 |
|  |  | positive | 0.681 | 0.014 | 0.701-0.724 |
|  |  | **Grade** |  |  |  |
|  |  | 1 | 0.321 | 0.071 | 0.361-0.496 |
|  |  | 2 | 0.718 | 0.064 | 0.709-0.832 |
|  |  | 3 | 0.719 | 0.035 | 0.656-0.716 |
|  |  | **Stage** |  |  |  |
|  |  | 1 | 0.607 | 0.2 | 0.299-0.672 |
|  |  | 2 | 0.772 | 0.034 | 0.716-0.78 |
|  |  | 3 | 0.769 | 0.058 | 0.725-0.829 |
|  |  | **ER/HER2** |  |  |  |
|  |  | ER-/HER2- | 0.752 | 0.04 | 0.736-0.805 |
|  |  | HER+ | 0.681 | 0.014 | 0.701-0.724 |
|  |  | ER+/HER2- | 0.802 | 0.08 | 0.742-0.887 |
|  | **ICpredictor** | **Age** |  |  |  |
|  |  | >54 yrs | 0.841 | 0.037 | 0.807-0.871 |
|  |  | ≤54 yrs | 0.769 | 0.056 | 0.665-0.76 |
|  |  | **ER** |  |  |  |
|  |  | negative | 0.764 | 0.023 | 0.732-0.775 |
|  |  | positive | 0.794 | 0.033 | 0.751-0.813 |
|  |  | **PR** |  |  |  |
|  |  | negative | 0.766 | 0.042 | 0.733-0.803 |
|  |  | positive | 0.735 | 0.015 | 0.612-0.638 |
|  |  | **HER2** |  |  |  |
|  |  | negative | 0.816 | 0.033 | 0.795-0.855 |
|  |  | positive | 0.734 | 0.024 | 0.752-0.797 |
|  |  | **Grade** |  |  |  |
|  |  | 1 | 0.5 | 0.074 | 0.47-0.605 |
|  |  | 2 | 0.796 | 0.043 | 0.802-0.876 |
|  |  | 3 | 0.771 | 0.038 | 0.707-0.775 |
|  |  | **Stage** |  |  |  |
|  |  | 1 | 0.67 | 0.253 | 0.375-0.85 |
|  |  | 2 | 0.811 | 0.027 | 0.762-0.813 |
|  |  | 3 | 0.848 | 0.032 | 0.83-0.886 |
|  |  | **ER/HER2** |  |  |  |
|  |  | ER-/HER2- | 0.752 | 0.04 | 0.736-0.805 |
|  |  | HER+ | 0.734 | 0.024 | 0.752-0.797 |
|  |  | ER+/HER2- | 0.842 | 0.035 | 0.82-0.881 |
| **Test set** | **Ipredictor** | **Age** |  |  |  |
|  |  | >44 yrs | 0.771 | 0.076 | 0.748-0.878 |
|  |  | ≤44 yrs | 0.727 | 0.048 | 0.733-0.818 |
|  |  | **Menopausal** |  |  |  |
|  |  | Pre- | 0.747 | 0.045 | 0.702-0.781 |
|  |  | Post- | 0.781 | 0.067 | 0.755-0.872 |
|  |  | **ER** |  |  |  |
|  |  | negative | 0.757 | 0.104 | 0.654-0.832 |
|  |  | positive | 0.704 | 0.105 | 0.59-0.79 |
|  |  | **PR** |  |  |  |
|  |  | negative | 0.771 | 0.071 | 0.647-0.777 |
|  |  | positive | 0.696 | 0.053 | 0.625-0.724 |
|  |  | **HER2** |  |  |  |
|  |  | negative | 0.785 | 0.014 | 0.759-0.785 |
|  |  | positive | 0.712 | 0.091 | 0.647-0.817 |
|  |  | **Ki67** |  |  |  |
|  |  | High | 0.736 | 0.009 | 0.766-0.783 |
|  |  | Low | 0.811 | 0.038 | 0.762-0.831 |
|  |  | **Grade** |  |  |  |
|  |  | 1 | 0.722 | 0.143 | 0.593-0.857 |
|  |  | 2 | 0.86 | 0.147 | 0.743-0.998 |
|  |  | 3 | 0.707 | 0.086 | 0.71-0.869 |
|  |  | **T stage** |  |  |  |
|  |  | 1 | 0.6 | 0.231 | 0.62-1 |
|  |  | 2 | 0.733 | 0.11 | 0.628-0.836 |
|  |  | 3 | 0.786 | 0.044 | 0.705-0.785 |
|  |  | **N stage** |  |  |  |
|  |  | 0 | 0.875 | 0.255 | 0.517-0.992 |
|  |  | 1-3 | 0.724 | 0.031 | 0.713-0.767 |
|  |  | **Stage** |  |  |  |
|  |  | 2 | 0.781 | 0.057 | 0.815-0.921 |
|  |  | 3 | 0.8 | 0.03 | 0.748-0.805 |
|  |  | **ER/HER2** |  |  |  |
|  |  | ER+/HER2- | 0.761 | 0.037 | 0.853-0.918 |
|  |  | ER-/HER2- | 0.739 | 0.092 | 0.682-0.85 |
|  |  | HER+ | 0.712 | 0.091 | 0.647-0.817 |
|  | **ICpredictor** | **Age** |  |  |  |
|  |  | >44 yrs | 0.85 | 0.007 | 0.836-0.849 |
|  |  | ≤44 yrs | 0.721 | 0.019 | 0.732-0.766 |
|  |  | **Menopausal** |  |  |  |
|  |  | Pre- | 0.733 | 0.071 | 0.666-0.784 |
|  |  | Post- | 0.847 | 0.043 | 0.835-0.912 |
|  |  | **ER** |  |  |  |
|  |  | negative | 0.7 | 0.102 | 0.626-0.812 |
|  |  | positive | 0.822 | 0.101 | 0.731-0.921 |
|  |  | **PR** |  |  |  |
|  |  | negative | 0.715 | 0.068 | 0.619-0.745 |
|  |  | positive | 0.833 | 0.062 | 0.746-0.857 |
|  |  | **HER2** |  |  |  |
|  |  | negative | 0.795 | 0.023 | 0.779-0.819 |
|  |  | positive | 0.695 | 0.091 | 0.626-0.799 |
|  |  | **Ki67** |  |  |  |
|  |  | High | 0.696 | 0.026 | 0.665-0.715 |
|  |  | Low | 0.92 | 0.037 | 0.856-0.922 |
|  |  | **Grade** |  |  |  |
|  |  | 1 | 0.75 | 0.14 | 0.561-0.825 |
|  |  | 2 | 0.699 | 0.124 | 0.634-0.839 |
|  |  | 3 | 0.768 | 0.056 | 0.711-0.815 |
|  |  | **T stage** |  |  |  |
|  |  | 1 | 0.7 | 0.4 | 0.22-0.98 |
|  |  | 2 | 0.789 | 0.109 | 0.669-0.875 |
|  |  | 3 | 0.734 | 0.017 | 0.663-0.694 |
|  |  | **N stage** |  |  |  |
|  |  | 0 | 0.958 | 0.096 | 0.842-1 |
|  |  | 1-3 | 0.778 | 0.038 | 0.756-0.819 |
|  |  | **Stage** |  |  |  |
|  |  | 2 | 0.802 | 0.131 | 0.64-0.879 |
|  |  | 3 | 0.802 | 0.05 | 0.78-0.876 |
|  |  | **ER/HER2** |  |  |  |
|  |  | ER+/HER2- | 0.807 | 0.073 | 0.847-0.976 |
|  |  | ER-/HER2- | 0.725 | 0.072 | 0.704-0.829 |
|  |  | HER+ | 0.695 | 0.091 | 0.626-0.799 |
