# Supplementary material for: Machine learning models based on immunological genes to predict the response to neoadjuvant therapy in breast cancer patients
Source: Front Immunol. 2022 Jul 22;13:948601. doi: 10.3389/fimmu.2022.948601 (PMC9352856; doi:10.3389/fimmu.2022.948601)
Supplement: Supplementary file 23 [file Table_11.docx]

**Supplementary Table 11.** AUROCs of the Ipredictor model, ICpredictor model, DLDA30 score, GGI, Onoctype DX score, and MammaPrint Score in the GSE20271 dataset

|  |  | **AUROC** | **Standard Deviation** | **95% Confidence Interval** |
| --- | --- | --- | --- | --- |
| **Entire set** | Ipredictor | 0.716 | 0.047 | 0.618-0.805 |
|  | ICpredictor | 0.752 | 0.048 | 0.648-0.839 |
|  | DLDA30 | 0.682 | 0.056 | 0.571-0.793 |
|  | GGI | 0.594 | 0.056 | 0.507-0.717 |
| **ER+/HER2- subset** | Ipredictor | 0.769 | 0.069 | 0.623-0.892 |
|  | ICpredictor | 0.782 | 0.076 | 0.62-0.922 |
|  | Onoctype DX | 0.543 | 0.065 | 0.504-0.743 |
|  | MammaPrint | 0.666 | 0.079 | 0.515-0.821 |
